# Elabela/Toddler Is an Endogenous Agonist of the Apelin APJ Receptor in the Adult Cardiovascular System, and Exogenous Administration of the Peptide Compensates for the Downregulation of Its Expression in Pulmonary Arterial Hypertension

**DOI:** 10.1161/CIRCULATIONAHA.116.023218

**Published:** 2017-03-20

**Authors:** Peiran Yang, Cai Read, Rhoda E. Kuc, Guido Buonincontri, Mark Southwood, Rubben Torella, Paul D. Upton, Alexi Crosby, Stephen J. Sawiak, T. Adrian Carpenter, Robert C. Glen, Nicholas W. Morrell, Janet J. Maguire, Anthony P. Davenport

**Affiliations:** From Experimental Medicine and Immunotherapeutics, University of Cambridge, Centre for Clinical Investigation, Addenbrooke’s Hospital, UK (P.Y., C.R., R.E.K., J.J.M., A.P.D.); Wolfson Brain Imaging Centre, Department of Clinical Neuroscience, University of Cambridge, UK (G.B., S.J.S., T.A.C.); Department of Pathology, Papworth Hospital, Papworth Everard, Cambridge, UK (M.S.); Centre for Molecular Informatics, Department of Chemistry, University of Cambridge, UK (R.T., R.C.G.); Department of Medicine, University of Cambridge, Addenbrooke’s Hospital, UK (P.D.U., A.C., N.W.M.); and Biomolecular Medicine, Department of Surgery and Cancer, Imperial College, London, UK (R.C.G.).

**Keywords:** apelin, cardiopulmonary, Elabela/Toddler, pulmonary hypertension, receptors, G-protein-coupled

## Abstract

**Background:**

Elabela/toddler (ELA) is a critical cardiac developmental peptide that acts through the G-protein–coupled apelin receptor, despite lack of sequence similarity to the established ligand apelin. Our aim was to investigate the receptor pharmacology, expression pattern, and in vivo function of ELA peptides in the adult cardiovascular system, to seek evidence for alteration in pulmonary arterial hypertension (PAH) in which apelin signaling is downregulated, and to demonstrate attenuation of PAH severity with exogenous administration of ELA in a rat model.

**Methods:**

*In silico* docking analysis, competition binding experiments, and downstream assays were used to characterize ELA receptor binding in human heart and signaling in cells expressing the apelin receptor. ELA expression in human cardiovascular tissues and plasma was determined using real-time quantitative polymerase chain reaction, dual-labeling immunofluorescent staining, and immunoassays. Acute cardiac effects of ELA-32 and [Pyr^1^]apelin-13 were assessed by MRI and cardiac catheterization in anesthetized rats. Cardiopulmonary human and rat tissues from PAH patients and monocrotaline- and Sugen/hypoxia-exposed rats were used to show changes in ELA expression in PAH. The effect of ELA treatment on cardiopulmonary remodeling in PAH was investigated in the monocrotaline rat model.

**Results:**

ELA competed for binding of apelin in human heart with overlap for the 2 peptides indicated by *in silico* modeling. ELA activated G-protein– and β-arrestin–dependent pathways. We detected ELA expression in human vascular endothelium and plasma. Comparable to apelin, ELA increased cardiac contractility, ejection fraction, and cardiac output and elicited vasodilatation in rat in vivo. ELA expression was reduced in cardiopulmonary tissues from PAH patients and PAH rat models, respectively. ELA treatment significantly attenuated elevation of right ventricular systolic pressure and right ventricular hypertrophy and pulmonary vascular remodeling in monocrotaline-exposed rats.

**Conclusions:**

These results show that ELA is an endogenous agonist of the human apelin receptor, exhibits a cardiovascular profile comparable to apelin, and is downregulated in human disease and rodent PAH models, and exogenous peptide can reduce the severity of cardiopulmonary remodeling and function in PAH in rats. This study provides additional proof of principle that an apelin receptor agonist may be of therapeutic use in PAH in humans.

## Abstract 2

Supplemental Digital Content is available in the text.

The apelin family of peptides interacts with a G-protein–coupled receptor named the apelin receptor or APJ. Apelin peptides have an emerging role in the adult cardiovascular system^1^ and in embryonic development of the heart,^2^ and [Pyr^1^]apelin-13 is the most abundant endogenous apelin peptide in the human heart.^3^ Alteration in the apelin system is thought to contribute to the etiology of cardiovascular diseases such as pulmonary arterial hypertension (PAH), a devastating disease with pulmonary vascular remodeling leading to death from right ventricular failure in which a beneficial effect of enhancing apelin receptor signaling has been proposed.^1^

Recently, 2 groups independently identified a well-conserved gene encoding a peptide named elabela (ELA)^4^ or toddler,^5^ required for early cardiac development in zebrafish. It is intriguing that the gene (*APELA*) was identified in a region not previously annotated as coding DNA. The *APELA* gene was predicted to express a 54-amino-acid protein comprising a 32-amino-acid mature peptide (ELA-32). Loss of this gene resulted in a rudimentary or no heart in fish embryos, a phenotype similar to loss of the gene *APLNR* encoding the apelin receptor. Both *APELA* and *APLNR* genes are expressed before gastrulation, whereas crucially the established ligand for this receptor, apelin (gene *APLN*), is not present and only expressed later in development. In agreement, deletion of the apelin gene did not produce the same phenotype as deletion of the apelin receptor gene, suggesting the presence of a second ligand such as ELA. In support, ELA was demonstrated to internalize the apelin receptor in vitro, and activation of the apelin signaling pathway was shown to rescue *APELA* mutants. It is interesting to speculate that ELA might be the first in a series of yet uncharacterized developmental signals.^4,5^ From these studies the existence of 3 peptides was proposed: ELA-32, ELA-21, and ELA-11 (Figure 1A).

**Figure 1.**
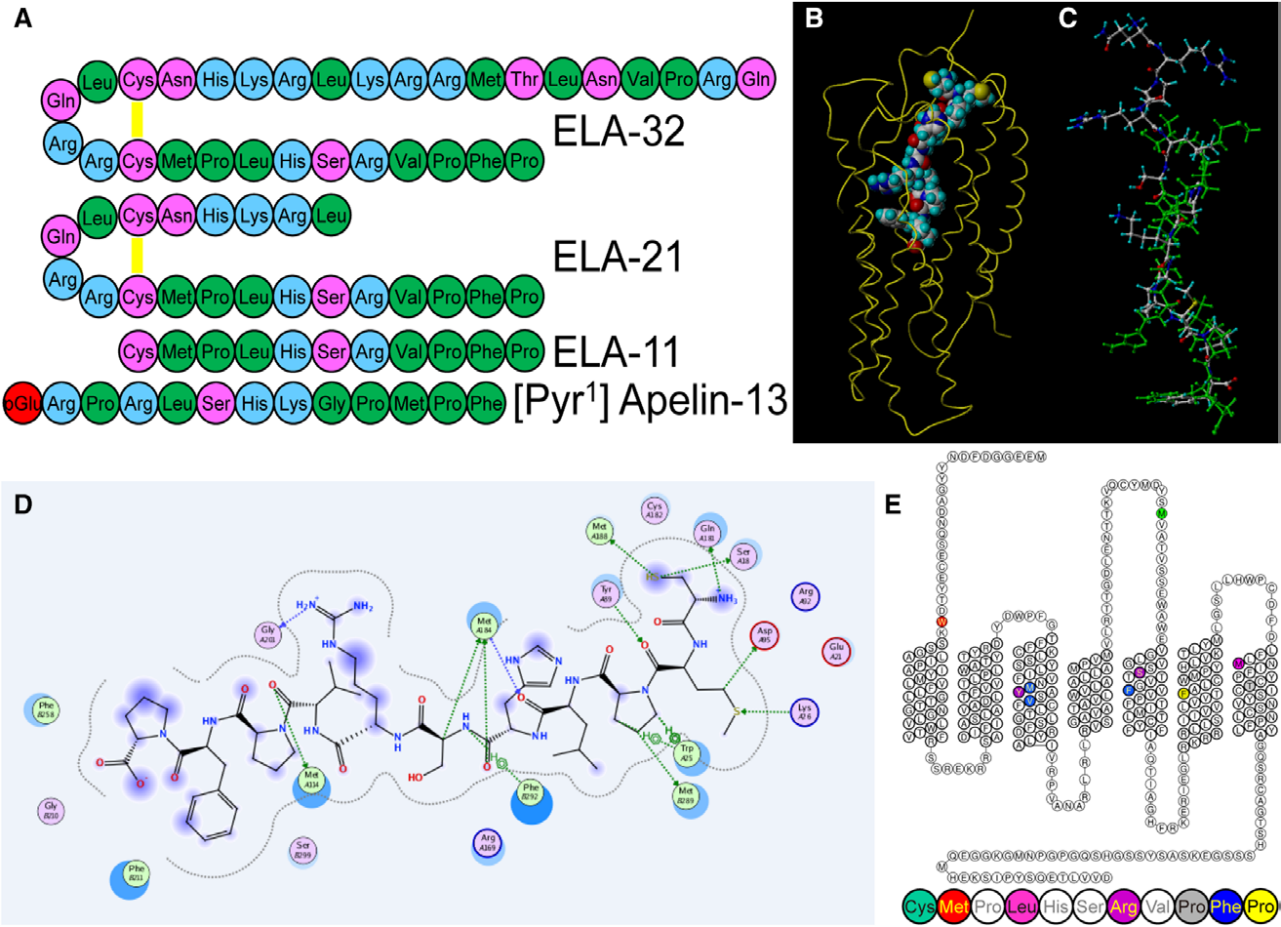
**ELA peptides bind to the human apelin receptor.** **A**, Amino acid sequences of ELA-32, ELA-21, ELA-11, and [Pyr^1^]apelin-13. Disulfide bridges are yellow lines, hydrophobic amino acids are shown in green, uncharged polar amino acids in pink, basic amino acids in blue with pyroglutamate in red. **B**, Docking of ELA-11 with the apelin receptor. **C**, Apelin-13 (multicolored atoms) and ELA-11 (green) docking showing overlap in the binding site. **D**, Ligand interaction diagram showing key close contacts between ELA-11 and the apelin receptor model. **E**, Predicted interactions of ELA-11 with the apelin receptor determined by docking analysis. Amino acids in the ELA-11 sequence are color coded to match their predicted sites of interaction on the receptor sequence. ELA indicates Elabela/toddler.

Our aim was to investigate the receptor pharmacology, expression pattern, and in vivo function of ELA peptides in the normal adult cardiovascular system and to seek evidence for alteration in PAH. Using *in silico* molecular modeling and docking, we propose a binding mode of ELA to the apelin receptor that is consistent with competition binding experiments, where the binding affinity of ELA was determined in human heart, a clinically relevant target. We have used cell-based pharmacological assays to show that ELA peptides activated the apelin receptor to inhibit cAMP production and to induce β-arrestin recruitment and receptor internalization. Results from real-time quantitative polymerase chain reaction experiments indicated the presence of *APELA* mRNA in human blood vessels with immunofluorescence staining, confirming the presence of mature ELA peptide localized to the vascular endothelium. ELA peptide was also detectable in human plasma. The acute cardiovascular effects of ELA in rat in vivo included increased cardiac contractility, ejection fraction and cardiac output, and, in addition, vasodilatation. Immunostaining and real-time quantitative polymerase chain reaction showed reduced expression in cardiopulmonary tissues from human PAH patients and 2 rat models of PAH, respectively. Crucially, we have demonstrated that ELA can attenuate the severity of changes in cardiopulmonary function/histology in the monocrotaline (MCT) rat model of PAH in vivo.

## Methods

The online-only Data Supplement Methods provides an expanded description of all experimental protocols. Human tissue samples were obtained with informed consent (Papworth Hospital Research Tissue Bank REC08/H0304/56) and local ethical approval (REC05/Q0104/142). All rodent experiments were performed according to the local ethics committee (University of Cambridge Animal Welfare and Ethical Review Body) and Home Office (UK) guidelines under the 1986 Scientific Procedures Act.

### Computational Methods

Docking of ELA-11 and the apelin receptor was conducted using the homology model of apelin and the GOLD docking algorithm as described previously.^6^

### Competition Binding

Experiments were conducted in homogenate of human ventricle or Chinese hamster ovary (CHO)-K1 cells expressing the human apelin receptor. Affinity (p*K*_i_) and receptor density (B_MAX_ fmol/mg) for ELA-32, ELA-21, and ELA-11 in human left ventricle (LV) were compared by 1-way analysis of variance (ANOVA), with Tukey multiple comparison. Data (p*K*_i_ and B_MAX_) for ELA-21 were compared between control LV and PAH LV and between control right ventricle (RV) and PAH RV by 2-tailed Student *t* test.

### Cell-Based Assays

Inhibition of cAMP accumulation, β-arrestin recruitment, and receptor internalization assays were used to determine values of potency (pD_2_ [–log_10_ EC_50_]) and maximum response (E_MAX_). pD_2_ values were compared by using 1-way ANOVA with Tukey multiple comparison.

Protein phosphorylation levels for an array of downstream signaling enzymes and levels of secreted angiogenic factors were determined by using pulmonary artery endothelial (PAECs) and smooth muscle cells treated with 0.1% serum, [Pyr^1^]apelin-13 or ELA-32 (both 100 nmol/L). Data were analyzed by 1-way ANOVA for matched data with Tukey multiple comparison.

### Real-Time Quantitative Polymerase Chain Reaction

RNA extraction, reverse transcription, and real-time quantitative polymerase chain reaction were performed as described in the online-only Data Supplement. For primer sequences, see online-only Data Supplement Table I.

### Immunostaining

Immunostaining was performed^7^ to localize ELA expression in human normal and PAH tissues using ELA antiserum that cross-reacted with ELA peptides, but not apelin (online-only Data Supplement Figure I), and to assess the effect of ELA-32 treatment on pulmonary vascular remodeling and cardiomyocyte hypertrophy in the MCT rat model as described in the online-only Data Supplement.

### Enzyme Immunoassays

Levels of ELA and apelin in healthy human plasma (n=25) were measured by using enzyme immunoassays compared using Student *t* test, and the correlation coefficient (Pearson *r*) was determined.

### MRI and Catheterization

The acute cardiac effects of ELA-32 and [Pyr^1^]apelin-13 were assessed by MRI and cardiac catheterization. MRI was performed in male Sprague Dawley rats (264±2 g) anesthetized with isoflurane (1.5%–2.5%, inhaled). Peak effects of ELA-32, [Pyr^1^]apelin-13, and saline on ejection fraction were expressed as change from baseline and compared by 1-way ANOVA with Dunnett post test.

In a second study, a pressure volume catheter was inserted to monitor LV hemodynamics in male Sprague Dawley rats (257±7 g) anesthetized with isoflurane (1.5% inhaled). Peak effects of ELA-32, [Pyr^1^]apelin-13, and saline on LV systolic pressure, cardiac output, stroke volume, contractility (dP/dt_MAX_), and heart rate were expressed as a change from baseline and compared by 1-way ANOVA with Dunnett post test.^8^

### MCT-Induced Rat Model of PAH

Male Sprague Dawley rats (205±2 g) were injected subcutaneously with MCT (60 mg/kg, n=18) or 0.9% saline (n=17) on day 0. MCT- (n=9) or saline (n=9)-exposed animals received daily intraperitoneal injections of ELA-32 (450 μg/kg) with the remainder (MCT, n=9; saline, n=8) receiving saline. On day 21, RV hemodynamics, RV hypertrophy, and pulmonary vascular remodeling were assessed and group data compared by using 1-way ANOVA with Tukey post test. The effect of chronic ELA administration on systemic blood pressure was investigated by LV catheterization in ELA control and saline control animals (n=5 each group) as described above.

### Statistical Analyses

Data are expressed as mean±standard error of the mean. Data and statistical analyses were conducted in GraphPad Prism 6. *P*≤0.05 was considered statistically significant.

## Results

### ELA Binds to the Apelin Receptor in Human Normal and PAH Heart

Structural alignment of ELA-11 and apelin-13 docked to the apelin receptor indicated they share a significant hydrophobic binding derived from the presence of the C-terminal hydrophobic moiety in a complimentary hydrophobic pocket of the apelin receptor. However, ELA-11 lacks positively charged residues directly corresponding to the important N-terminal RPRL motif in apelin-13 required for initial recognition of the peptide, although it is interesting to note that there are positively charged amino acids in this region in the longer ELA sequences (Figure 1A). We have recently described a possible pose of apelin-13 interacting with the apelin receptor cavity^6^; given the similarities of the C terminus, we assumed that the pose of this region might be comparable for ELA-11. It has been hypothesized that F10 on ELA-11 may assume a pose similar to F13 on apelin-13,^6^ and our model indicated that the binding cavity is large enough to accept the additional C-terminal hydrophobic proline (P11) of ELA-11. Following docking analysis, the pose that consistently showed the best GOLD docking score is shown in Figure 1B. ELA-11 and apelin-13 showed a high degree of overlap in the binding site (Figure 1C). The ELA-11 pose obtained after docking analysis showed a large number of interactions with the apelin receptor (Figure 1D), mainly involving the hydrophobic residues in the N-terminal and C-terminal section of the peptide (Figure 1E) but also including hydrogen bonds.

**Figure 2.**
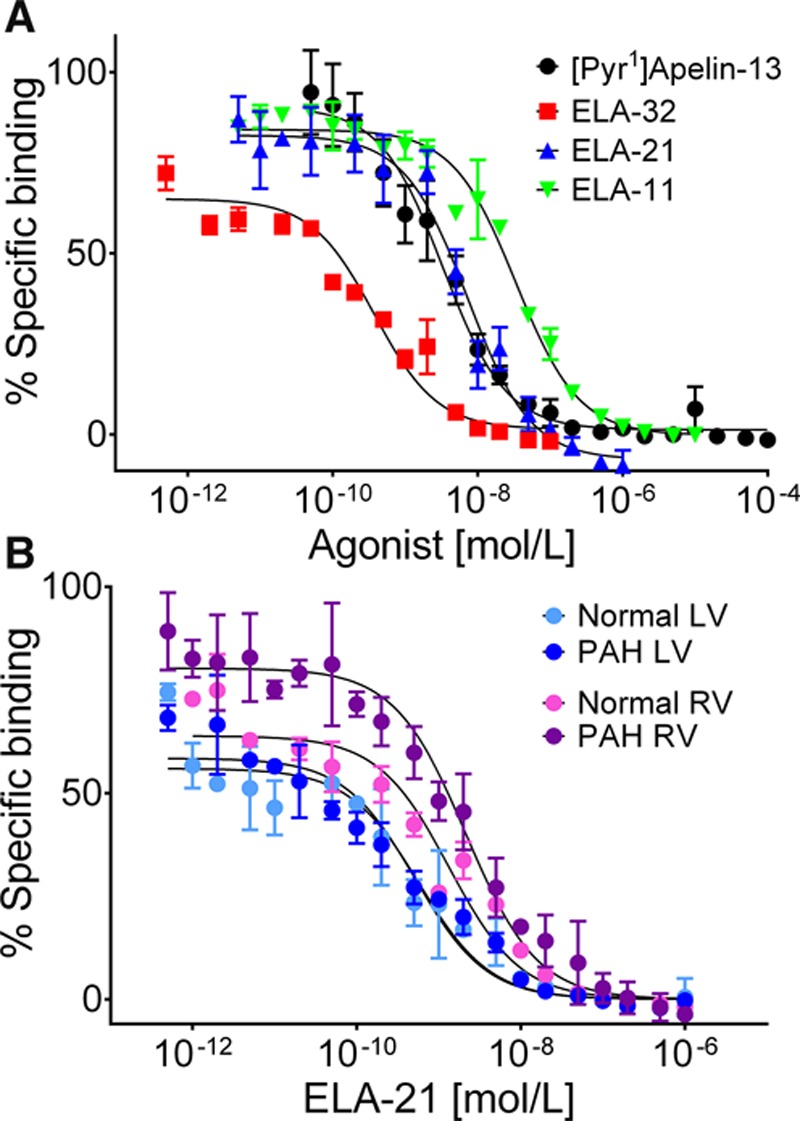
**Competition binding curves for ELA peptides in human heart.** **A**, ELA-32, ELA-21, ELA-11, and [Pyr^1^]apelin-13 in human left ventricle. **B**, ELA-21 in normal right ventricle, PAH right ventricle, normal left ventricle, and PAH left ventricle. Values are mean±SEM, n=3. ELA indicates Elabela/toddler; PAH, pulmonary arterial hypertension; and SEM, standard error of the mean.

In human LV ELA-32 (p*K*_i_=9.59±0.08) had significantly (*P*≤0.0001) higher affinity than ELA-21 (p*K*_i_=8.52±0.11) and [Pyr^1^]apelin-13 (p*K*_i_=8.85±0.04), which were comparable. In contrast, ELA-11 (p*K*_i_=7.85±0.05) had significantly lower affinity than the other peptides (*P*≤0.01) (Figure 2A) suggesting that a longer sequence with positively charged residues in the N terminus is required for optimal binding affinity. This is supported by data for the extended peptide ELA-14 that competed for binding in CHO-K1 cells with subnanomolar affinity (p*K*_i_=9.35±0.02), whereas cyclo[1–6]ELA-11 had lower affinity, as expected (p*K*_i_=7.27±0.03).

ELA-21 bound with comparable affinities in RV and LV from human normal and PAH hearts (RV, normal p*K*_i_=8.98±0.04, PAH p*K*_i_=9.30±0.07; LV, normal p*K*_i_=9.31±0.11, PAH p*K*_i_=9.46±0.10) (Figure 2B). In PAH heart in comparison with control, there was a small (≈15%) but significant reduction in apelin receptor density in both LV (PAH 3.42±0.15 fmol/mg, normal 3.96±0.08 fmol/mg; *P*≤0.05) and RV (PAH 3.5±0.05 fmol/mg, normal 4.24±0.03 fmol/mg, *P*≤0.001).

### Receptor Pharmacology of ELA in Vitro

**Figure 3.**
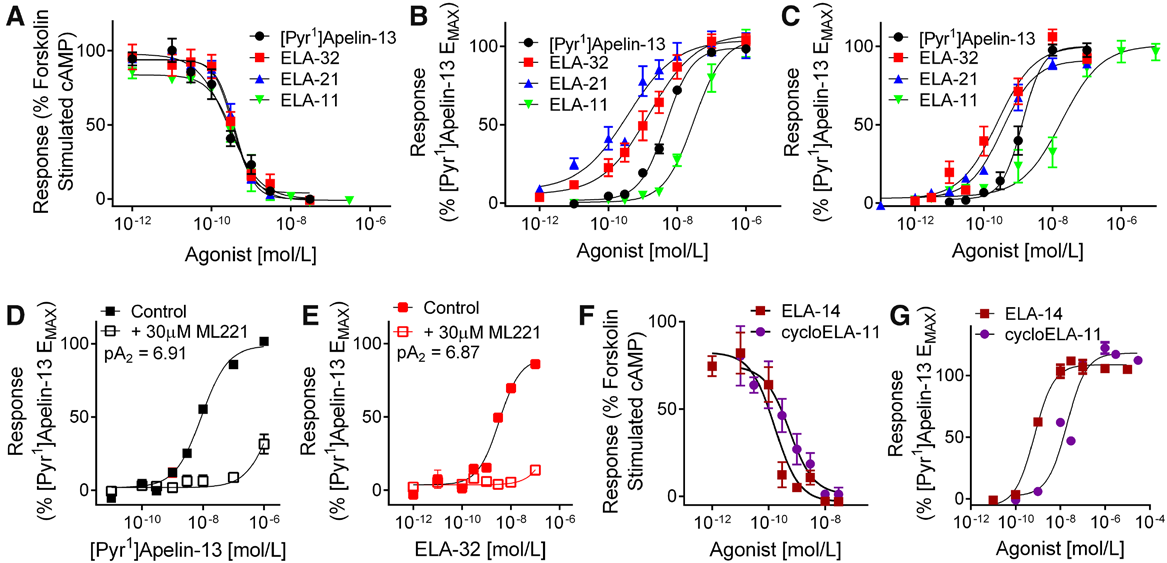
**Concentration-response curves in cell-based receptor pharmacology assays.** **A**, Inhibition of forskolin-induced cAMP accumulation. **B**, Stimulation of β-arrestin recruitment. **C**, Induction of receptor internalization. [Pyr^1^]apelin-13, ELA-32, ELA-21, and ELA-11. Antagonism of [Pyr^1^]apelin-13 (**D**) and ELA-32 (**E**) by 30 μmol/L ML221 in the β-arrestin assay. Concentration-response curves to ELA-14 and cyclic ELA-11 in cAMP (**F**) and β-arrestin (**G**) assays. Values are mean±SEM, n=3 to 5 assays, 2 to 6 replicates per assay. ELA indicates Elabela/toddler; and SEM, standard error of the mean.

ELA-32, ELA-21, ELA-11, and [Pyr^1^]apelin-13 completely inhibited forskolin-induced cAMP production in a concentration-dependent manner (Figure 3A) with subnanomolar potencies (Table). The potency of ELA-11 was comparable to the longer ELA peptides, indicating that this short sequence retains full biological activity in this G-protein–coupled assay.

**Table.**
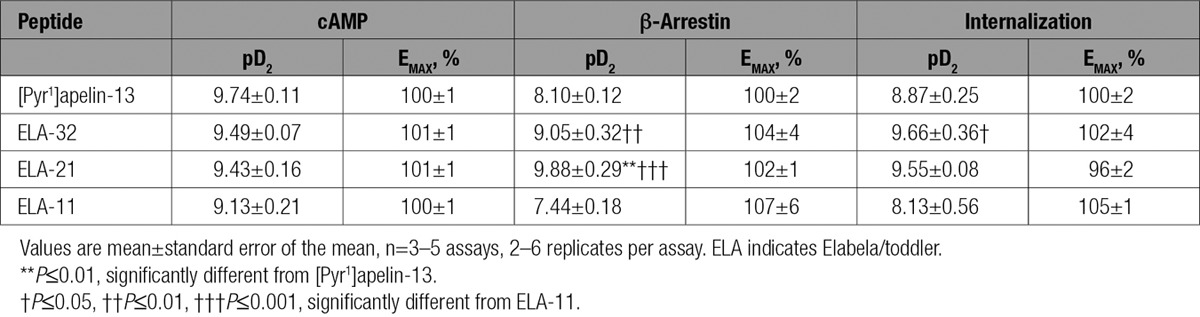
Potency (pD2) and Efficacy (EMAX) of [Pyr1]apelin-13, ELA-32, ELA-21, and ELA-11 in cAMP Inhibition, β-Arrestin Recruitment, and Receptor Internalization Assays

ELA-32, ELA-21, ELA-11, and [Pyr^1^]apelin-13 stimulated β-arrestin recruitment in a concentration-dependent manner (Figure 3B) with similar efficacy. ELA-32 and ELA-21 exhibited comparable pD_2_ values and were significantly more potent than ELA-11. A similar rank order of potency was obtained in the internalization assay (Figure 3C), suggesting that a longer sequence is required for optimal activation of the β-arrestin pathway (Table). ML221 (Tocris) antagonized the concentration-response curves to ELA-32 (Figure 3D) and [Pyr^1^]apelin-13 (Figure 3E) in the β-arrestin recruitment assay with comparable pA_2_ values of 6.87 and 6.91, respectively. ELA-14 (pD_2_=10.09±0.12) and cyclo[1–6]ELA-11 (pD_2_=9.42±0.32) exhibited subnanomolar potency in the cAMP assay (Figure 3F) similar to the other ELA peptides. In β-arrestin, ELA-14 (pD_2_=9.08±0.04) was equipotent with ELA-32 and cyclo[1–6]ELA-11 (pD_2_=7.67±0.02) had potency comparable to ELA-11 (Figure 3G).

In control PAECs and control and PAH pulmonary artery smooth muscle cells, apelin and ELA-32 increased levels of ERK1/2 phosphorylation, and in PAECs there was also a significant increase in phosphorylation of endothelial nitric oxide synthase (online-only Data Supplement Figure IIA through IID). Phosphorylation levels of other kinases (online-only Data Supplement Figure III) and levels of secreted angiogenic factors (online-only Data Supplement Figures IV and V) were unaffected.

### ELA Is Expressed in Human Cardiovascular Tissues

**Figure 4.**
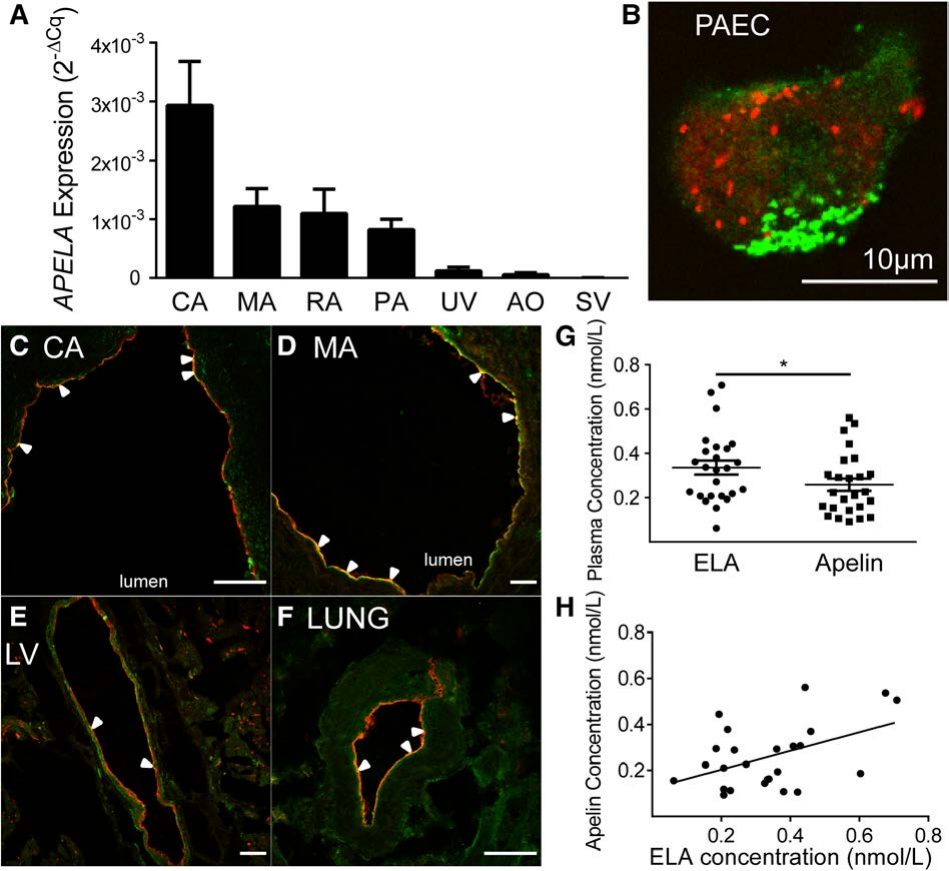
**Expression of *APELA* transcript and ELA peptide in human cardiovascular tissues.** **A**, Expression of *APELA* mRNA in human blood vessels determined by RT-qPCR. **B** through **F**, Representative overlay confocal photomicrographs of immunofluorescence staining of human pulmonary artery endothelial cell (PAEC) (**B**) and human cardiovascular tissue (**C** through **F**). Photomicrographs show ELA-like immunoreactivity in green and the endothelial marker vWF in red. If not indicated, scale bars=75 μm. CA, coronary artery (n=11); MA, mammary artery (n=6); RA, radial artery (n=9); PA, pulmonary artery (n=4); UV, umbilical vein (n=6); AO, aorta (n-6); SV, saphenous vein (n=8); LV, left ventricle (n=8); lung (n=6). **G**, Levels of ELA and apelin in human plasma (n=25). **H**, Correlation between plasma concentrations of ELA and apelin. *P<0.05 in comparison with ELA. ELA indicates Elabela/toddler; RT-qPCR, real-time quantitative polymerase chain reaction; and vWF, von Willebrand factor.

Expression of *APELA* transcript was observed in all human blood vessels investigated (Figure 4A). With the exception of the aorta, there was a trend for *APELA* expression to be higher in arteries than in veins. Lower levels were detectable in heart and lung (not shown).

Punctate staining of ELA-like immunoreactivity (-LI) and von Willebrand factor-LI were observed in human PAECs but not colocalized to the same intracellular vesicles (Figure 4B). ELA-LI was found in the intima of coronary (Figure 4C) and mammary arteries (Figure 4D), and colocalized with von Willebrand factor. Endothelial ELA-LI was detectable in blood vessels in the heart (Figure 4E) and lung (Figure 4F, online-only Data Supplement Figure VI). No ELA-LI was observed in smooth muscle cells or cardiomyocytes.

ELA and apelin were detectable in healthy human plasma at 0.34±0.03 nmol/L and 0.26±0.03 nmol/L, respectively (Figure 4G), with significantly higher levels of ELA than apelin (*P*≤0.05). There was a weak, but significant (*P*≤0.05), positive correlation between ELA and apelin peptide levels (Pearson’s *r*=0.47) (Figure 4H).

### Cardiovascular Effects of ELA in Rodents in Vivo

**Figure 5.**
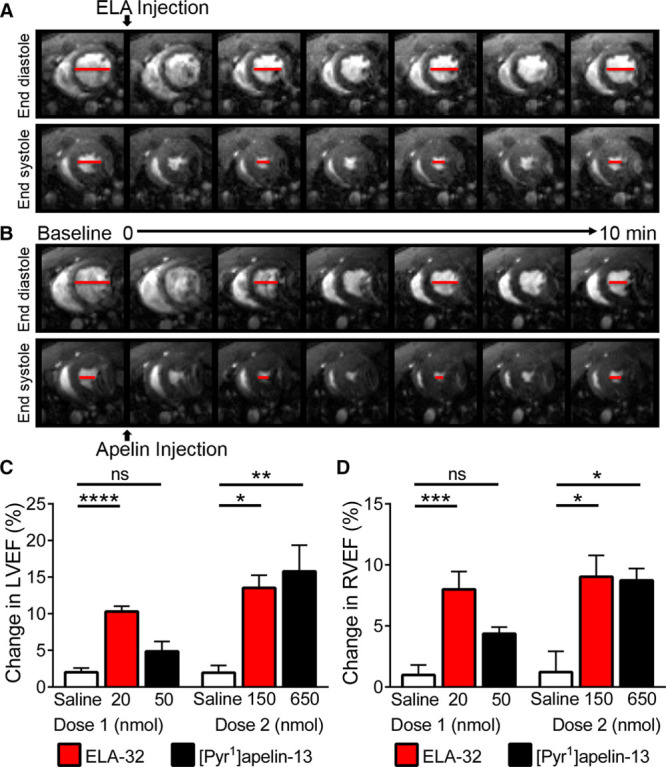
**Cardiac effects of ELA and apelin in vivo by MRI in the rat.** Representative snapshots of midventricular transverse sections of the heart at end-diastolic and end-systolic points during the 10-minute MRI scan showing the effects of ELA-32 (150 nmol) (**A**) and [Pyr^1^]apelin-13 (650 nmol) (**B**). Red bars are drawn to show the approximate diameter of the left ventricle. Dose-dependent increase in left (**C**) and right (**D**) ventricular ejection fraction of the heart in response to ELA-32 (red bars, 20 and 150 nmol, n=8) and [Pyr^1^]apelin-13 (black bars, 50 and 650 nmol, n=5). *P<0.05. **P<0.01. ***P<0.001. ****P<0.0001 in comparison with saline control (n=6). ELA indicates Elabela/toddler; LVEF, left ventricular ejection fraction; ns, not significantly different from saline control; and RVEF, right ventricular ejection fraction.

ELA-32 and [Pyr^1^]apelin-13 directly strengthened cardiac contractions in vivo, as illustrated in the representative MRI videos (online-only Data Supplement Movies I and II) and snapshots (Figure 5A and 5B). ELA-32 and [Pyr^1^]apelin-13 dose-dependently increased LV and RV ejection fractions (Figure 5C and 5D). Low-dose ELA-32 (20 nmol) and [Pyr^1^]apelin-13 (50 nmol) increased ejection fraction by 10.3±0.7% (*P*≤0.0001) and 4.9±1.4% (*P*>0.05) in LV, respectively, in comparison with saline control (2.0±0.6%) and by 8.0±1.5% (*P*≤0.001) and 4.4±0.5% (*P*>0.05) in RV, respectively, in comparison with saline control (1.0±0.8%). High-dose ELA-32 (150 nmol) and [Pyr^1^]apelin-13 (650 nmol) significantly increased ejection fraction in LV by 13.5±1.7% (*P*≤0.05) and 15.8±3.6% (*P*≤0.01), respectively, in comparison with saline control (1.9±1.0%), and in RV by 9.0±1.8% (*P*≤0.05) and 8.7±1.0% (*P*≤0.05), respectively, in comparison with saline control (1.2±1.7%). Effects were short acting and returned to baseline after 10 minutes for the low dose, but were still present at 10 minutes for the high dose. ELA-32 appeared more potent than [Pyr^1^]apelin-13, because lower doses of ELA-32 were able to achieve equivalent or more pronounced effects than [Pyr^1^]apelin-13.

**Figure 6.**
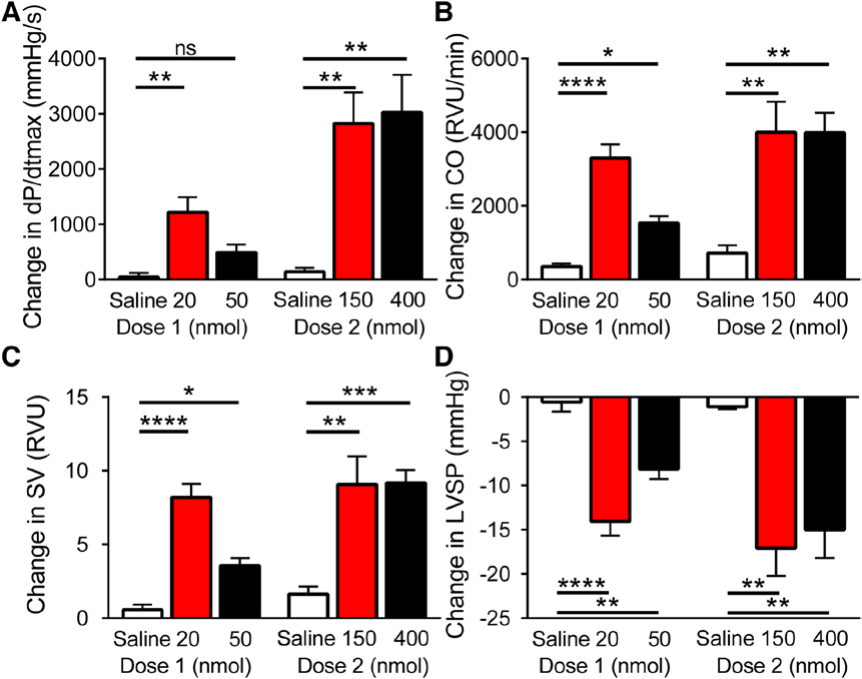
**The in vivo effects of ELA-32 and [Pyr^1^]apelin-13 on the left ventricle of rat heart measured by catheterization.** Compared to saline controls (open bars, n=6), ELA-32 (red bars, n=10), and [Pyr^1^]apelin-13 (black bars, n=8) caused a significant change in cardiac contractility (dP/dt_MAX_) (**A**), cardiac output (CO) (**B**), stroke volume (SV) (**C**), and LV systolic pressure (LVSP) (**D**). *P<0.05. **P<0.01. ***P<0.001. ****P<0.0001 in comparison with saline control. ELA indicates Elabela/toddler; LV, left ventricular; RVU, relative volume unit; and ns, not significantly different from saline control.

In the second rat study, ELA-32 (20 nmol) and [Pyr^1^]apelin-13 (50 nmol) caused a rapid increase in dP/dt_MAX_ of 1217±272 mm Hg/s (*P*≤0.01) and 493±144 mm Hg/s (*P*>0.05) from baseline, respectively, in comparison with saline control (49±71 mm Hg/s). At the higher dose, ELA-32 (150 nmol) and [Pyr^1^]apelin-13 (400 nmol) significantly increased dP/dt_MAX_ by 2825±565 mm Hg/s (*P*≤0.01) and 3025±680 mm Hg/s (*P*≤0.01), respectively, in comparison with saline control (142±69 mm Hg/s) (Figure 6A). This effect on contractility coincided with a significant increase in cardiac output at both doses of ELA-32 (20 nmol, 3296±370 relative volume units/min (RVU/min), *P*≤0.0001; 150 nmol, 4000±826 RVU/min, *P*≤0.01) and [Pyr^1^]apelin-13 (50 nmol, 1535±189 RVU/min, *P*≤0.05; 400 nmol, 3989±537 RVU/min, *P*≤0.01) in comparison with saline control (352±86 RVU/min; 716±215 RVU/min) (Figure 6B). The increase in cardiac output was mostly attributable to an increased stroke volume, because end-systolic volume was reduced. Stroke volume was also significantly increased by ELA-32 (20 nmol, 8.2±0.9 RVU, *P*≤0.0001; 150 nmol, 9.1±1.9 RVU, *P*≤0.01) and [Pyr^1^]apelin-13 (50 nmol, 3.6±0.5 RVU, *P*≤0.05; 400 nmol, 9.2±0.9 RVU, *P*≤0.001) in comparison with saline control (0.6±3.4 RVU, 1.6±0.5 RVU) (Figure 6C). A trend toward increased heart rate for both peptides did not reach statistical significance (online-only Data Supplement Figure VII). This effect was followed by a significant dose-dependent reduction in LV systolic pressure by ELA-32 (20 nmol, 14.1±1.6 mm Hg, *P*≤0.0001; 150 nmol, 17.1±3.2 mm Hg, *P*≤0.01) and [Pyr^1^]apelin-13 (50 nmol, 8.2±1.1 mm Hg, *P*≤0.01; 400 nmol, 8.2±3.2 mm Hg, *P*≤0.01), in comparison with the saline controls (0.6±1.1 mm Hg; 1.1±0.3 mm Hg) (Figure 6D). A third, higher dose of [Pyr^1^]apelin-13 (1300 nmol) did not show further increase in any response. To confirm the contribution of systemic vasodilatation to the reduction in LV systolic pressure, ELA-32 and [Pyr^1^]apelin-13 were each administered to 1 animal in which the catheter was placed in the carotid artery and both elicited a comparable reduction in systolic and diastolic blood pressure (not shown). Consistent with the MRI experiment, the cardiovascular effects of ELA-32 and [Pyr^1^]apelin-13 were short acting, returning to baseline within 10 to 20 minutes. Moreover, the response to ELA-32 appeared to be greater than the same dose of [Pyr^1^]apelin-13.

### Downregulation of ELA Expression in Human PAH and Rodent Models of PAH

**Figure 7.**
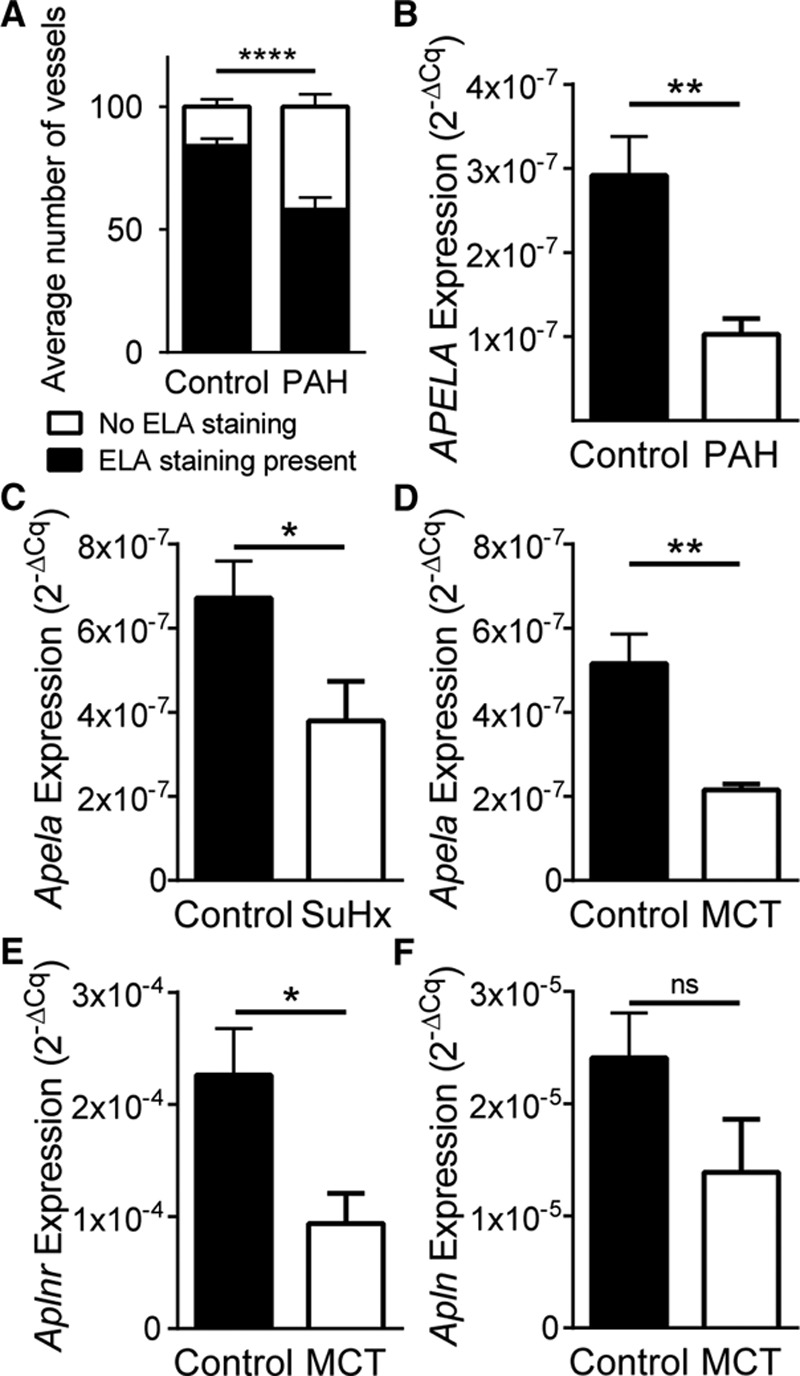
**Altered levels of ELA expression in PAH**. **A**, Number of blood vessels positive and negative for ELA staining in control (n=4) and PAH (n=4) human lung sections (****P≤0.0001). **B**, *APELA* downregulation in human PAH (n=4) in comparison with normal (n=4) lung (**P≤0.01). *Apela*, mRNA downregulation in the right ventricle of Sugen/hypoxia (n=6) (**C**) and MCT exposed (n=5) (**D**) rats (*P≤0.05, **P≤0.01, respectively, in comparison with saline (n=7 and 5, respectively). *Aplnr* (**E**) and *Apln* mRNA (**F**) expression in right ventricle of saline (n=5) and MCT exposed (n=5) rats (*P≤0.05, in comparison with saline. ELA indicates Elabela/toddler; MCT, monocrotaline; ns, not significantly different from saline control; and PAH, pulmonary arterial hypertension.

The number of ELA-positive and ELA-negative blood vessels in control human lung sections was 84±3 versus 16±3, in comparison with 58±5 versus 42±5 in PAH lung (Fisher exact test, *P*≤0.0001; Figure 7A). In comparison with controls, the proportion of ELA-positive vessels was significantly reduced, whereas the proportion of ELA-negative vessels was significantly increased in PAH (online-only Data Supplement Figure VIII).

Expression of *APELA* mRNA in human PAH lung was significantly (*P*≤0.01) reduced in comparison with healthy lung (Figure 7B). *Apela* mRNA level in RV was significantly reduced in both Sugen/hypoxia (*P*≤0.05) and MCT rats (*P*≤0.01) in comparison with controls (Figure 7C and 7D). It is interesting to note that *Aplnr* mRNA in RV was also significantly lower in the MCT rats than in controls (*P*≤0.01) (Figure 7E), and there was a trend (not significant, *P*=0.14) for reduced expression of *Apln* mRNA in these animals (Figure 7F).

### Attenuation of MCT-Induced PAH by ELA

**Figure 8.**
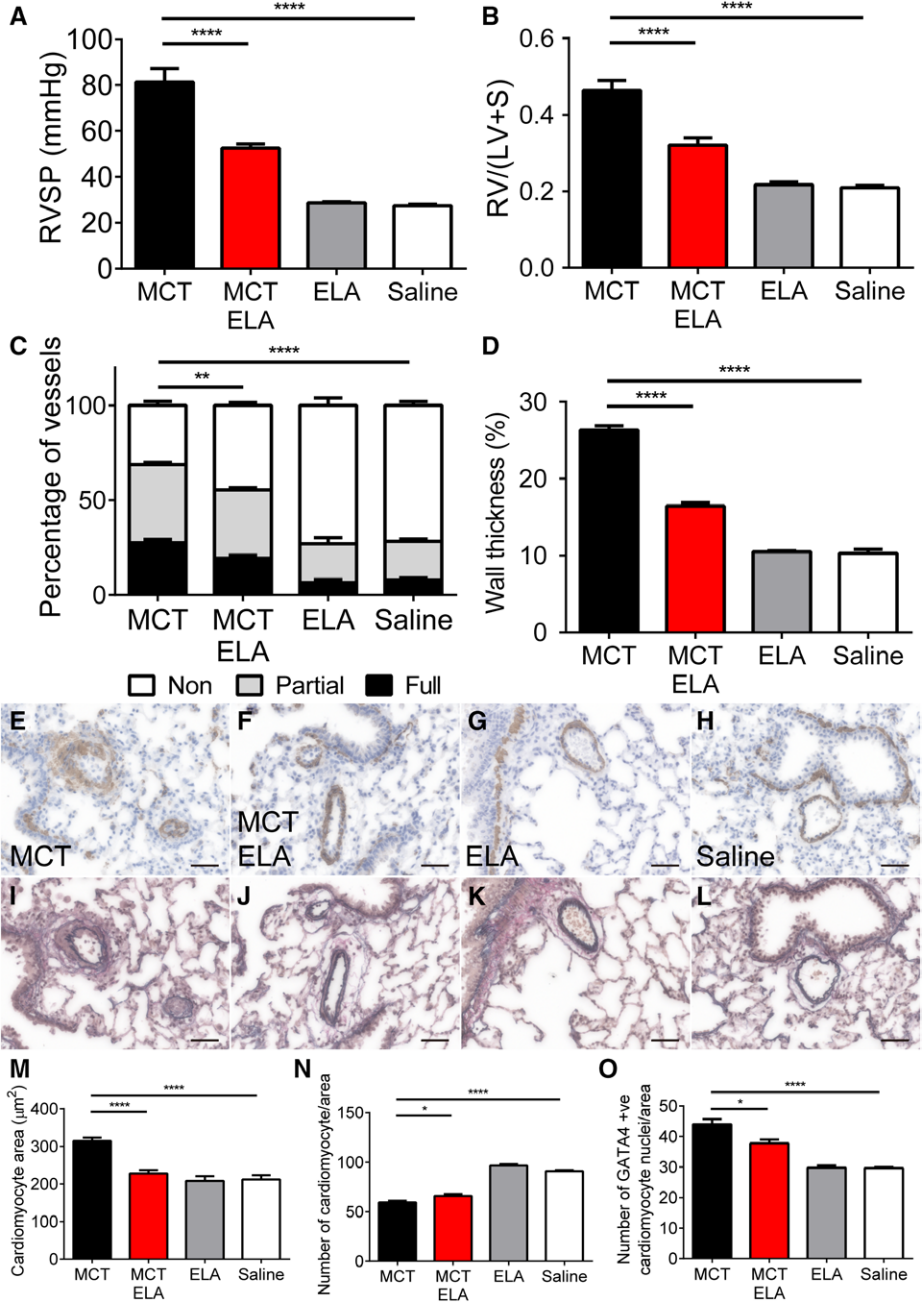
**Attenuation of MCT-induced PAH by ELA-32 in rats.** MCT exposure (black bar, n=9) caused an increase in right ventricular systolic pressure (RVSP) (**A**) and right ventricular hypertrophy (**B**) measured as RV/(LV+S) (Fulton index) (both ****P≤0.0001) in comparison with saline control (open bars, n=8). ELA-32 administration (red bars, n=9) significantly reduced MCT-induced RVSP and hypertrophy (****P≤0.0001 and ****P≤0.0001, respectively). ELA-32 alone (gray bars, n=9) had no effect and was not different from saline control (open bars, n=8). In tissues from these animals, MCT increased the proportion of fully muscularized vessels in rat lung (**C**) and wall thickness of larger pulmonary arterioles (**D**) in comparison with saline (both ****P≤0.0001), and these changes were attenuated by ELA-32 (**P≤0.01 and ****P≤0.0001, respectively). Immunohistological visualization of remodeling of pulmonary arterioles using α-smooth muscle actin (brown, **E** through **H**) and van Giesen stain (**I** through **L**) in sections of lung from MCT (**E**, **I**), MCT-ELA (**F**, **J**), ELA alone (**G**, **K**), and saline control rats (**H**, **L**) (scale bars=75μm). MCT-induced RV hypertrophy was indicated by an increase in cardiomyocyte area indicated by WGA staining (**M**), a reduction in cardiomyocyte number/area (**N**), and an increase in GATA4-positive nuclei/area compared to saline control (**O**) (****P≤0.0001) with a significant improvement in these indices following ELA-32 treatment (*P≤0.05, **** *P*≤0.0001, in comparison with MCT alone). ELA-32 alone had no effect on any parameter. ELA indicates Elabela/toddler; MCT, monocrotaline; LV, left ventricle; ns, not significantly different from saline control; PAH, pulmonary arterial hypertension; RV, right ventricle; and WGA, wheat germ agglutinin.

MCT administration elevated RV systolic pressure (81.3±6.0 mm Hg, *P*≤0.0001) in comparison with saline controls (27.4±0.6 mm Hg). ELA-32 treatment in MCT rats attenuated RV systolic pressure (52.5±1.9 mm Hg, *P*≤0.0001 versus MCT group), but this was still significantly higher than control RV systolic pressure (*P*≤0.0001 versus saline group) (Figure 8A). Similarly, in comparison with the saline controls (Fulton index=0.21±0.01), RV hypertrophy was observed in MCT rats (0.46±0.02, *P*≤0.0001) that was significantly attenuated by ELA-32 administration (0.32±0.02, *P*≤0.0001 versus MCT group, *P*≤0.05 versus saline group) (Figure 8B). MCT increased the percentage of fully muscularized vessels (MCT 28±2% versus saline 8±1%, *P*≤0.0001) and arteriolar wall thickness (MCT 26±1% versus saline 10±1%, *P*≤0.0001), and this was significantly lessened by ELA-32 (muscularization, 19±2%, *P*≤0.01 versus MCT; wall thickness, 16±1% versus MCT, *P*≤0.0001) (Figure 8C through 8L). MCT exposure resulted in significant RV hypertrophy indicated by an increase in cardiomyocyte area (wheat germ agglutinin staining; MCT 315±9 μm^2^ versus saline 212±11 μm^2^, *P*≤0.0001), a reduction in cardiomyocyte number/area (MCT 59±2 versus saline 91±1, *P*≤0.0001) and an increase in GATA4-positive nuclei/area (MCT 44±2 versus saline 30±1, *P*≤0.0001). There was a significant improvement in these indices following ELA-32 treatment (cardiomyocyte area 228±9 μm^2^, *P*≤0.0001 in comparison with MCT alone; cardiomyocyte number/area 66±2; GATA4 positive nuclei/area 38±1 (both *P*≤0.05 in comparison with MCT alone) (Figure 8M through 8O, online-only Data Supplement Figure IX). ELA-32 had no significant effect in comparison with saline control on any hemodynamic or histological parameter in this study and, in particular, did not cause systemic hypotension following chronic administration (online-only Data Supplement Figure X). There was some evidence of improved survival in the MCT-ELA group in comparison with MCT alone, because in the MCT group 1 rat died (day 18) and 3 died under isoflurane, whereas only 1 MCT-ELA rat died (day 20). Plasma levels of the vasoactive peptides angiotensin-II and BNP-32 were unaltered by ELA-32 treatment (online-only Data Supplement Figure XI).

## Discussion

This study provides a comprehensive characterization of ELA, originally reported as a regulator of zebrafish cardiac development, in the adult mammalian cardiovascular system. We report for the first time ELA peptides binding to the native human apelin receptor, ELA expression in human blood vessels, attenuation of cardiac dysfunction, cardiac and pulmonary arterial remodeling, and peptide and receptor mRNA expression in human PAH and rodent models of PAH, in addition to more fully characterizing the cardiovascular profile of ELA in comparison with apelin.

### Receptor Binding and Downstream Signaling

Using cells overexpressing the receptor and ELA conjugated to alkaline phosphatase, Chng et al^4^ were the first to suggest that ELA could bind to the human apelin receptor. In this study, we initially used molecular dynamic simulation based on homology models of the apelin receptor to create a receptor template into which ELA could be reliably docked. Despite the lack of obvious sequence similarity, ELA-11 docked within the binding pocket occupied by apelin-13. We subsequently confirmed this in radioligand competition assays using [^125^I]apelin-13 to demonstrate direct binding of all 3 ELA peptides to the apelin receptor in human cardiac tissue. Our data from both heart and CHO cells revealed that ELA-32, ELA-21, and ELA-14 bound to the human receptor with subnanomolar affinity, whereas the linear and cyclo[1–6]ELA-11 displayed a 100-fold drop in affinity, consistent with a recent report using HEK293 cells expressing the apelin receptor.^9^ This confirms the importance of positively charged amino acids (which interact via hydrogen bonding with the apelin receptor in our model system) in the longer ELA peptides corresponding to the RPRL motif in apelin-13 that we have previously identified as critical for receptor-binding affinity.^10^ Similar or lower affinities for ELA conjugated to alkaline phosphatase have been reported in CHO cells expressing apelin receptor (*K*_d_=0.51 nmol/L^11^) and for ELA-32 in isolated rat cardiomyocytes (*K*_i_=38.2 nmol/L^12^). Therefore, although a recent report from the codiscoverers of ELA suggested an unidentified cell surface receptor rather than the apelin receptor is responsible for mediating the effects of ELA in human embryonic stem cells,^13^ our data clearly confirm that in the adult cardiovascular system ELA is a ligand for the apelin receptor.

Next, ELA was tested for its ability to activate G-protein signaling using a cAMP inhibition assay, because the apelin receptor is known to couple via G_i_. ELA-32, ELA-21, and ELA-11 were full agonists in this assay with comparable potency to [Pyr^1^]apelin-13 in the subnanomolar range, consistent with their high binding affinities. It is interesting to note that ELA-11 and cyclo[1–6]ELA-11 were not different from the longer forms in terms of potency and efficacy in blocking cAMP accumulation, suggesting that the 11-mer was sufficient for effective G_i_-protein signaling via the apelin receptor. ELA-14, consistent with our high-affinity binding data, has also been reported to activate G-protein–dependent and –independent pathways with comparable potency to ELA-32,^9^ and our data confirm this. Data for ELA-32 have been reported by 2 other groups with EC_50_ values in the subnanomolar^11^ to nanomolar^14^ range and ELA-mediated inhibition of cAMP production confirmed as pertussis toxin sensitive.^11^

As expected for agonist activation of most G-protein–coupled receptors, apelin binding also triggered recruitment of β-arrestin leading to receptor internalization and β-arrestin–dependent signaling.^15^ Our data revealed that, in contrast to their equivalent potency in the G-protein pathway, ELA-32, ELA-21, and ELA-14 were more potent than ELA-11, cyclo[1–6]ELA-11, and [Pyr^1^]apelin-13 in the β-arrestin assays. These data are interesting because they provide additional evidence that extended N-terminal sequences of ELA and apelin peptides are more effective in stimulating β-arrestin–mediated cellular events as previously reported for apelin-17 in comparison with [Pyr^1^]apelin-13 in cAMP and internalization assays using the expressed rat receptor.^16^ Our data expand on initial studies that demonstrated internalization of enhanced green fluorescent protein–tagged apelin receptors in zebrafish embryos by exogenous *APELA* mRNA and ELA-21 peptide^5^ and ELA-32–induced internalization of green fluorescent protein–tagged human apelin receptor overexpressed in HEK293 cells.^14^ The nonpeptide small-molecule antagonist ML221 blocked responses to ELA-32 and [Pyr^1^]apelin-13 in the β-arrestin assay with the same affinity, providing additional evidence that the 2 ligands bind to the same or overlapping sites on the receptor. It is important to note that for drug discovery and therapeutic intervention, these data confirm that apelin receptor antagonists can be designed that will block all apelin signaling irrespective of the endogenous ligand. We conclude from our cell-based assays that the truncated form, ELA-11, preferentially activates G-protein signaling and may represent an endogenous G-protein–biased apelin receptor ligand. How the different apelin and ELA peptides are integrated in normal apelin receptor physiology and how these may contribute to disease progression remain to be unraveled.

### ELA Expression in the Human Cardiovascular System

Having established receptor binding and activation by ELA peptides in vitro, we addressed the question of endogenous expression of ELA in relevant human tissues. To date, *APELA* mRNA expression has been reported in human embryonic stem cells,^4,14^ induced pluripotent stem cells,^14^ kidney^4,14^ and prostate,^4^ and rat kidney^11^ with ELA peptide expression only reported in human embryonic stem cells.^4^ Our study is the first report of *APELA* transcript and ELA in human blood vessels suggesting that *APELA* is translated into a peptide in the vasculature. Expression was identified in both large- and small-diameter vessels; for example, in heart, ELA localized to both epicardial and intramyocardial blood vessels. Specifically, ELA peptide expression was restricted to the vascular endothelium with no significant localization to vascular smooth muscle or cardiomyocytes. We have previously reported the localization of the apelin receptor to the vascular endothelium, the underlying smooth muscle/cardiomyocytes,^17^ raising the possibility that ELA may signal in an autocrine/paracrine manner. We have also localized apelin to the vascular and endocardial endothelium,^7^ and therefore an overlapping distribution of the 2 peptides is apparent. The significance of 2 peptides and 1 receptor will need to be addressed; however, in development this is resolved by temporal differences in the expression of each peptide. The expression of *APELA* appeared to be higher in arterial than in venous tissue, with the exception of the aorta. The implication of this trend is not clear. The presence of ELA peptide in the heart was consistent with previous reports on zebrafish, where ELA is critical in cardiac development,^4,5^ and Perjés et al^12^ recently reported *APELA* mRNA expression in endothelial cells in mouse heart. Staining of cultured primary human endothelial cells verified the presence of ELA peptide in this cell type. It is interesting to note that ELA did not colocalize with Weibel-Palade bodies,^18^ suggesting that ELA is produced via a constitutive synthetic pathway rather than via regulated/inducible release. This subcellular spatial localization is also observed with apelin.^17^

Apelin levels have been measured in human plasma by enzyme-linked immunosorbent assay.^19^ We therefore used a similar assay to detect ELA peptides. Both ELA and apelin were detectable in human plasma at subnanomolar levels, more indicative of peptides acting as locally released autocrine/paracrine mediators than as circulating hormones. Although we observed a correlation between plasma concentrations of ELA and apelin, the relatively narrow age range of our samples did not allow a more detailed correlation between ELA concentration and, for example, age, sex, or body mass index.

### Cardiovascular Effect of ELA in Rodents in Vivo

We next tested if ELA modulates cardiovascular functions in vivo. In heart, apelin is reportedly the most potent inotrope in vitro^3,20^ via protein kinase C and extracellular signal–regulated kinases 1/2.^21^ Consistent with this are data from in vivo studies in rodents and humans. In anesthetized rats, apelin-16 increased dP/dt_MAX_^22^ and [Pyr^1^]apelin-13 increased cardiac output and reduced blood pressure.^6^ In anesthetized mice, intraperitoneal injection of [Pyr^1^]apelin-13 reduced LV end-diastolic area and increased heart rate, observed using MRI, whereas hemodynamics measurement by catheterization showed an increased preload recruitable stroke work (a measure of intrinsic contractility) and reduced LV end-systolic pressure.^23^ In human volunteers, intracoronary bolus administration of apelin-36 increased dP/dt_MAX_, and an intravenous infusion of apelin-36 or [Pyr^1^]apelin-13 increased heart rate and cardiac output.^24^

We have used a combination of MRI and invasive catheterization to characterize the cardiac effects of ELA in comparison with apelin. ELA-32 and [Pyr^1^]apelin-13 were positive inotropes in the LV, increasing dP/dt_MAX_ consistent with the previous reports for apelin^22,24^ and an initial report of increased fractional shortening by ELA-32.^9^ In addition, ELA-32 and [Pyr^1^]apelin-13 increased cardiac output, owing to increased stroke volume and possibly increased heart rate. We also observed for ELA-32 the previously reported apelin-induced reduction in LV end-diastolic area,^23^ resulting in an increased ejection fraction. We could not detect obvious qualitative differences in the cardiac actions of ELA-32 and [Pyr^1^]apelin-13, but Perjés et al^12^ reported that the inotropic effect of ELA-32 in vitro in the Langendorff perfused rat heart was dependent on extracellular signal–regulated kinases 1/2 but not on protein kinase C as seen for apelin.

We detected an ELA-32– and [Pyr^1^]apelin-13–induced drop in LV systolic pressure. This is likely a consequence of reduced afterload resulting from peripheral vasodilatation, a known effect of apelin.^3,6^ To confirm systemic vasodilation, ELA-32 and [Pyr^1^]apelin-13 were administered with the catheter placed in the right carotid artery, and both peptides caused a drop in blood pressure in agreement with Murza et al^9^. These in vivo data are consistent with recent in vitro studies suggesting that ELA has a vasodilatory effect in adult rat coronary arteries^12^ and caused relaxation of preconstricted mouse aortic rings^14^ in a reported nitric oxide–independent and partially endothelium-independent manner. Overall, we observed that lower doses of ELA-32 were required to achieve equivalent or more pronounced effects than [Pyr^1^]apelin-13. This is consistent with the ≈5-fold higher receptor affinity we determined for ELA-32 in comparison with [Pyr^1^]apelin-13 in human heart.

### Reduced ELA Expression in Human PAH and Rodent Models and Attenuation of MCT-Induced PAH by Exogenous ELA in Rats

Last, we addressed the question of the possible alteration in ELA expression in PAH, where apelin expression is known to be reduced, contributing to disease pathogenesis. Apelin levels are downregulated in plasma or serum^19,25^ in PAECs^26^ and pulmonary microvascular endothelial cells^27^ from PAH patients and in RV of MCT-exposed rat.^28^ The expression of the apelin receptor was also reduced in RV of MCT rats.^28^ We have now shown for the first time that ELA is similarly reduced in pulmonary vessels of PAH patients and in the RV from 2 rodent models of PAH. In particular, ELA staining was examined in small pulmonary blood vessels that are critical to the pathogenesis of PAH, because they undergo vascular remodeling leading to increased vascular resistance and, consequently, the RV undergoes hypertrophy. We observed a reduction in *Apela* expression in RV of Sugen/hypoxia and MCT-exposed rats and also of *Aplnr* with a trend to reduction in *Apln* in the MCT animals. This is consistent with a downregulation of all components of the ELA/apelin/apelin receptor pathway in the RV in PAH. It is important to note that, although reduced, *Aplnr* mRNA was still present in the RV, making the receptor amenable for therapeutic manipulation with a goal to replace the downregulated ELA or apelin peptides. We confirmed this in human heart where the apelin receptor density in PAH in comparison with normal RV and LV was only reduced by ≈15%. There has been 1 study reporting increased *Apela* and *Aplnr* mRNA expression in LV of a mouse model of myocardial infarction,^12^ but we did not observe this for the receptor in LV from human PAH heart. Last, we have shown that, as reported for apelin,^28^ administration of ELA-32 attenuated the remodeling of the pulmonary vasculature and hypertrophy of RV cardiomyocytes. This resulted in blunting of the increased RV systolic pressure and hypertrophy induced by MCT in this rat model of PAH.

In conclusion, our study confirms the direct receptor binding of ELA to the apelin receptor in human heart, and provides more details on the docking of this ligand within the receptor using molecular modeling. Using cell-based assays, we compared ELA-32, ELA-21, and ELA-11 with [Pyr^1^]apelin-13 and found them to be agonists in G-protein–dependent and –independent pathways. Our data also demonstrated the widespread presence of *APELA* mRNA and ELA peptide in adult human cardiovascular tissues and localized the peptide specifically to the endothelium. Furthermore, we demonstrated in vivo that ELA increases cardiac contractility and cardiac output and causes vasodilatation. These results show that ELA is an endogenous agonist of the human apelin receptor and exhibits a cardiovascular profile comparable to that of apelin. The relative importance of the 2 peptides to normal apelin receptor function needs to be explored. However, the downregulation of ELA expression in PAH and the beneficial effect of ELA administration on cardiac function and cardiopulmonary remodeling in the MCT rat model of PAH, consistent with that of apelin, supports the potential exploitation of the apelin receptor as a therapeutic target at least in this disease.

## Acknowledgments

The authors thank Keith Siew for technical advice and discussion, the theater and consultant staff of Papworth hospital for tissue collection, and Dr Benjamin Garfield for rat tissues.

## Sources of Funding

This work was supported by the Wellcome Trust 107715/Z/15/Z and Program in Metabolic and Cardiovascular Disease 096822/Z/11/Z, Medical Research Council MC_PC_14116, British Heart Foundation PS/02/001, PG/05/127/19872, FS/14/59/31282, and, in part, by the National Institute for Health Research Cambridge Biomedical Research Center and the Pulmonary Hypertension Association UK.

## Disclosures

None.

## Supplementary Material

## Clinical Perspective

### What Is New?

- Elabela/toddler (ELA) was first identified as an essential peptide in the development of the heart in zebrafish, and proposed as a second endogenous ligand at the apelin receptor.
- We have now shown that ELA is widely expressed in the adult human cardiovascular system, localizing to the vascular endothelium and plasma.
- ELA peptides activated G-protein– and β-arrestin–dependent pathways with comparable potency to apelin and, crucially, these actions were blocked by apelin receptor antagonists.
- ELA increased cardiac contractility, ejection fraction, and cardiac output, and it elicited vasodilatation in anesthetized rats in vivo.

### What Are the Clinical Implications?

- Apelin is known to be downregulated in human and animal models of pulmonary arterial hypertension (PAH).
- We have demonstrated that ELA did not compensate for the loss of apelin, because its expression was also significantly reduced in cardiopulmonary tissues from human PAH patients and in rat models of PAH.
- Treatment with exogenous ELA attenuated right ventricular hypertrophy, systolic pressure, and pulmonary vascular remodeling in the monocrotaline rat model of PAH.
- The results suggest that a selective first-in-class agonist that mimics the action of the endogenous ligands apelin/ELA is a promising therapeutic strategy in the treatment of PAH distinct from pathways targeted by current clinical treatments.
