# Supplementary material for: Elabela/Toddler Is an Endogenous Agonist of the Apelin APJ Receptor in the Adult Cardiovascular System, and Exogenous Administration of the Peptide Compensates for the Downregulation of Its Expression in Pulmonary Arterial Hypertension
Source: Circulation. 2017 Mar 20;135(12):1160–73. doi: 10.1161/CIRCULATIONAHA.116.023218 (PMC5363837; doi:10.1161/CIRCULATIONAHA.116.023218)
Supplement: Supplementary file 3 [file cir-135-1160-s003.pdf]

SUPPLEMENTAL MATERIAL

Elabela/Toddler is an endogenous agonist of the apelin APJ receptor in the adult cardiovascular system, and exogenous administration of the peptide compensates for the downregulation of its expression in pulmonary arterial hypertension

First author's surname: Yang

Short title: Down-regulation of Elabela/Toddler in PAH

Peiran Yang[1] MA, Cai Read[1] MRes, Rhoda E Kuc[1] BA, Guido Buonincontri[2] PhD, Mark Southwood[3] PhD, Rubben Torella[4] PhD, Paul D Upton[5] PhD, Alexi Crosby[5] PhD, Stephen J Sawiak[2] PhD, T Adrian Carpenter[2] PhD, Robert C Glen[4,6] PhD, Nicholas W Morrell[5] MD, Janet J Maguire*[1] PhD, Anthony P Davenport*[1] PhD

[1]Experimental Medicine and Immunotherapeutics, University of Cambridge, Level 6, Centre for Clinical Investigation, Box 110, Addenbrooke's Hospital, Cambridge, CB2 0QQ, U.K. [2]Wolfson Brain Imaging Centre, Department of Clinical Neuroscience, University of Cambridge, Box 65, CB2 0QQ, Cambridge, U.K. [3]Department of Pathology, Papworth Hospital, Papworth Everard, Cambridge, CB23 8RE, U.K. [4]Unilever Centre for Molecular Sciences Informatics, Department of Chemistry, University of Cambridge, Lensfield Road, Cambridge, CB2 1EW, U.K. [5]Department of Medicine, University of Cambridge, Box 157, Addenbrooke's Hospital, Cambridge, CB2 0QQ, U.K. [6]Biomolecular Medicine, Department of Surgery and Cancer, Imperial College, London, SW7 2AZ, U.K.

†The new address of G.B. is Istituto Nazionale di Fisica Nucleare, Sezione di Pisa, Edificio C, Largo Bruno Pontecorvo, 3, 56127, Pisa, Italy.

Corresponding author: Dr. Anthony Davenport

Experimental Medicine and Immunotherapeutics,

University of Cambridge,

Level 6, Centre for Clinical Investigation,

Box 110, Addenbrooke’s Hospital,

Cambridge, CB2 0QQ, UK.

Phone: +44 (0)1223 336899

Fax: +44 (0)1223 761576

Email: apd10@medschl.cam.ac.uk

* Joint last authors

Supplemental Methods

Human tissues samples were obtained with informed consent (Papworth Hospital Research Tissue Bank REC08/H0304/56) and local ethical approval (REC05/Q0104/142). All rodent experiments were performed according to the local ethics committee (University of Cambridge Animal Welfare and Ethical Review Body) and Home Office (UK) guidelines under the 1986 Scientific Procedures Act.

Comptutational Methods

Molecular dynamics simulation of ELA-11 binding to the apelin receptor was conducted as previously described[1]. Briefly, the modelling template was based on a modified 2.5 Å resolution crystal structure of the human CXCR4 chemokine receptor (PDB code 3ODU[2]). MODELLER 9v8[3] was used for generating homology models of apelin. ELA-11 has been computationally designed using the SCHRÖDINGER software suite[4]. The apelin receptor and ELA-11 were used as a starting point for further docking analysis, using GOLD v5.1[5, 6]. An in-house script was created to create a constraint between the side-chains of F257 and W261 in the apelin receptor (predicted to interact with the C-terminal Phe of apelin and critical for receptor internalization[7]) and F10 in ELA-11 that greatly reduced the search space for docking, resulting in a pose consistent with current mutagenesis data[8]. No other constraints were applied to the complex. Images and diagrams were made to show peptide docking in the binding site using Pymol (Schrödinger, LLC) and key close contacts ELA-11 and the apelin receptor[9].

Human Tissue Collection

Human tissue samples were collected with local ethical approval and informed consent and were frozen and stored at -70°C until use. Left ventricle (LV) and RV was from six normal

donor hearts for which there were no suitable recipients and LV was additionally obtained from fourteen patients transplanted for cardiomyopathy. LV and right ventricle (RV) was from six patients with pulmonary arterial hypertension (PAH). Histologically normal lung tissue and pulmonary artery were from patients undergoing lobectomy, additional lung tissue was collected from four patients with PAH. Coronary artery and aorta were collected from dilated cardiomyopathy patients. Saphenous vein, left internal mammary artery and radial artery were from patients receiving coronary artery bypass graft surgery. Human plasma samples were collected from healthy volunteers.

Comptetition Binding Experiments

For structure activity studies competition binding experiments were conducted in triplicate in homogenate of pooled human LV from cardiomyopathy patients as previously described[1]. Homogenate of human LV was incubated for 90 min with 0.1nmol/L [Glp^{65},Nle^{75},Tyr^{77}][^{125}I]apelin-13 in assay buffer (mmol/L: Tris 50, $MgCl_2$ 5, pH 7.4, 22ºC), in the presence of increasing concentrations of human ELA-32, ELA-21, ELA-11 or [Pyr^{1}]apelin-13 peptides (0.5pmol/L-10μmol/L) (n=3 each). Non-specific binding was defined using 1μmol/L [Pyr^{1}]apelin-13. Equilibrium was broken by centrifugation (20,000g for 10 min, 4ºC). Pellets were washed with Tris-HCl buffer (50 mmol/L, pH 7.4, 4 ºC), re-centrifuged and bound radioactivity in final pellets counted. Data from triplicate experiments were analyzed using the iterative non-linear curve fitting programs EBDA and LIGAND (KELL package, Biosoft, UK) or GraphPad Prism6 and to derive values of affinity (expressed as the $-\log_{10}$ of the dissociation constant (pK_i ±sem)) and receptor density (B_{MAX}±sem). The pK_i and B_{MAX} values for the three putative endogeous forms of ELA (ELA-32, ELA-21 and ELA-11) were compared using ANOVA with Tukey's post tests. The synthetic analogues ELA-14 and cyclo[1-6]ELA-11 were tested for affinity at the human apelin receptor expressed in CHO-K1 cells (data obtained from Cerep, Celle L'Evescault, France).

To investigate whether the affinity of ELA peptides or the density of ELA binding sites is altered in human PAH competition binding experiments were repeated as described above using ELA-21 as the competing ligand in LV and RV from pooled homogenate from six patients transplanted for PAH and LV and RV from six normal hearts as controls. Data from triplicate experiments were analyzed using the iterative non-linear curve fitting programs EBDA and LIGAND (KELL package, Biosoft, UK) to determine values of affinity (K_D) and receptor density (B_{MAX}). Values were compared for LV and RV between PAH and controls using Student's 2-tailed *t*-test.

Inhibition of cAMP Accumulation, β-Arrestin Recruitment and Receptor Internalisation Assays

Second messenger signalling and receptor pharmacology were studied in assays (DiscoveRx, Fremont, CA, USA) according to instructions from the manufacturer and as previously described[1]. 3-5 assays with 2-6 replicates each were performed.

For inhibition of forskolin-induced cAMP accumulation, CHO-K1 cells artificially expressing the human apelin receptor were seeded in Cell Plating medium into 96-well plates and incubated for 24 hours at 37^oC in 5% CO_2, followed by replacement of the medium with cAMP Antibody Reagent in Cell Assay Buffer. Basal levels of cAMP were elevated by 15μmol/L forskolin, which was incubated with the cells for 30 minutes at 37°C, in the absence or presence of human ELA-32, ELA-21, ELA-11, [Pyr^1]apelin-13, or two synthetic analogues, ELA-14 and cyclo[1-6]ELA-11, (1pmol/L-0.3μmol/L) diluted in Cell Assay Buffer. Cells were incubated with a mixture of Lysis Buffer, cAMP Buffer D and Detection Reagents for 1 hour incubation at room temperature, followed by a 3 hour incubation with cAMP Reagent A at room temperature, and chemiluminescence reading (LumiLITE™ Microplate Reader,

DiscoveRx). Responses measured in relative light units were fitted to 4 parameter logistic concentration response curves in GraphPad Prism 6 (La Jolla, CA, USA) and values of potency, pD_2 ($-\log_{10} EC_{50}$ (where EC_{50} is the concentration producing half maximal response)), and maximum response (E_{MAX}) were calculated for each compound. Data were subsequently expressed normalised as percentage inhibition of forskolin-stimulated cAMP production.

β-Arrestin assays were conducted as previously described[1]. CHO-K1 cells artificially expressing the human apelin receptor were seeded in Cell Plating medium into 96-well plates and incubated for 48 hours at 37°C in 5% CO_2. Human ELA-32, ELA-21, ELA-11, [Pyr[1]]apelin-13, or two synthetic analogues, ELA-14 and cyclo[1-6]ELA-11, (1pmol/L-3μmol/L) were diluted in Cell Plating medium and added to the cells for 90 minutes at 37°C. . For antagonist experiments, additional 30-minute incubation was carried out prior to addition of agonists with 30μmol/L ML221[10] (Tocris Bioscience, Bristol, UK) made in Cell Plating medium. Detection reagents were then added for a 2-hour incubation at room temperature followed by chemiluminescence reading. Responses measured in relatve light units were fitted to 4 parameter logistic concentration response curves in GraphPad Prism 6 (La Jolla, CA, USA) and values of pD_2 ($-\log_{10} EC_{50}$) and maximum response (E_{MAX}) were calculated for each compound. Data were subsequently normalized to the maximum response to [Pyr[1]]apelin-13 used as the reference agonists in each assay. For antagonist experiments using a small molecule antagonist ML221[10], antagonist affinities, pA_2 ($-\log K_B$, where K_B is the antagonist dissociation constant) were determined for the apelin receptor.

Internalisation assays were conducted as previously described[1]. U2OS cells artificially expressing the human apelin receptor were seeded in Cell Plating medium in 96-well plates and incubated for 48 hours at 37°C in 5% CO_2. Human ELA-32, ELA-21 or ELA-11, or [Pyr[1]]apelin-13 (0.1pmol/L-10μmol/L) were diluted in Cell Plating medium and incubated with

the cells for 3 hours at 37°C. This was followed by incubation with the detection reagents for 90 minutes at room temperature and luminescence reading. Data were analysed as described for the β-arrestin assay.

Protein Phosphorylation and Angiogenesis Assays in Cultured PAEC and PASMC

Control pulmonary artery endothelial cells (PAECs, n=3) (Lonza) and control (n=3) and PAH (n=3) pulmonary artery smooth muscle cells (PASMCs) (from sex matched controls and patients with BMPRII mutations) were plated at 330,000/6cm dish and allowed to adhere. Cells were serum starved (0.1% fetal bovine serum) overnight, washed in PBS and subsequently treated for 10 minutes with either 0.1% serum (control) or [Pyr^{1}]apelin (100nmol/L) or ELA-32 (100nmol/L). Cells were then washed in PBS, lysed at 4°C for 30 minutes, centrifuged at 12,000g for 7.5 mins and lysates stored at -70°C prior to the assay. The relative levels of protein phosphorylation for an array of 43 kinase phosphorylation sites and 2 related total proteins were determined in duplicate using lysates (100ng protein per well) of the treated cells according to the manufacturer's instructions (Proteome Profiler Array, Human Phospho-Kinase Array Kit, Catalogue No. ARY003B, R&D Systems Inc. Minneapolis, USA). The assay was performed as directed by the manufacturer and membranes exposed for 3 minutes. Spot density was quantified using Image J and the average determined for the duplicate data. The appropriate negative control value was subtracted from all readings and data expressed as arbitary units (AU). Data were analysed by one-way ANOVA for repeated (matched) measures with Tukey's post test for multiple comparisons.

For angiogenesis assays, cells were plated at 60,000/well in 12 well plates. The conditioned media from the the same cells treated for 24 hours with 0.1% serum, [Pyr^{1}]apelin or ELA-32 (both 100nmol/L) was used to determine the relative levels of 55 secreted human angiogenesis-related proteins according to the manusfacturer's instructions (Proteome Profiler Array, Human

Angiogenesis Array Kit, Catalogue No. ARY007, R&D Systems Inc. Minneapolis, USA). Data were quantified and analysed as described for the phospho-kinase assay.

APELA mRNA Expression by Reverse Transcription and qPCR

The mRNA expression of *APELA* (gene for ELA) was studied in human cardiovascular tissues using reverse transcription and quantitative real-time PCR. For RNA extraction, cubes ($5mm^3$), or the equivalent amount, of human coronary (n=11), mammary (n=6), radial (n=9), and pulmonary artery (n=4), aorta (n=6), umbilical (n=6) and saphenous vein (n=6), LV (n=8), lung (n=6) were incubated with TRIzol® Reagent (Life Technologies, Paisley, UK) in metal bead lysing matrix (Lysing Matrix D for heart and lung tissues) (MP Biomedicals, Santa Ana, CA, USA), and homogenized using the FastPrep-24™ 5G system (MP Biomedicals) for up to 6 runs at 6.5m/s for 45 seconds. After homogenization, total RNA was extracted using PureLink™ RNA Mini Kit (Life Technologies) with DNase treatment included, performed according to the manufacturer's instructions. The yield of RNA was determined with NanoDrop 1000 spectrophotometer (Wilmington, DE, USA) and 1μg of RNA from each sample was used for reverse transcription with the Promega Reverse Transcription System (Promega, Madison, WI, USA), carried out according to manufacturer's instructions. The cDNA product was used in triplicates for real-time quantitative PCR was performed for 45 cycles using the ABI 7500 Real-Time PCR System (Life Technologies) with double-dye Taqman primer probes for human *APELA* gene from Primerdesign (Chandlers Ford, UK) and for human 18S rRNA (Life Technologies) as the internal control. The primer sequences or IDs are shown in Supplemental Table 1. The expression of *APELA* was normalised to that of 18S using the comparative C_q method[11].

Alteration in *APELA* mRNA expression in pulmonary arterial hypertension (PAH) was studied in lungs from three PAH and one pulmonary veno-occlusive disease (class 1' PAH) patients

and tissue from four histologically normal lungs. For comparison expression was also determined in tissue from two rodent models of PAH. Rat tissue from monocrotaline (MCT) treated animals was generously provided by Dr Benjamin Garfield (Imperial College London). Male Sprague-Dawley rats (208±3g) was given a subcutaneous injection of MCT (40mg/kg body weight) to induce PAH, or PBS (phosphate buffered saline) as controls (n=5 each). The animals were sacrificed by terminal anaesthesia and exsanguination three weeks after MCT injection and the heart was removed. Tissue from Sugen/hypoxia exposed (n=7) and weight matched control (n=6) animals was a kind gift from Emily Groves (Morrell Group, University of Cammbridge). Male Sprague-Dawley rats (150-200g) were given a subcutaneous injection of Sugen 5416 (20 mg/kg, Tocris, Bristol, UK), housed in hypoxic chambers at 10% O_2 for 3 weeks, followed by 8 weeks normoxia prior to euthanasia by exsanguination and removal of the heart. mRNA expression in the RV of these rats were compared with weight-matched controls. The RV was used to study *Apela*, *Aplnr* and *Apln* (genes for ELA, apelin receptor and apelin) mRNA expression, determined as desribed above using rat 18S rRNA as internal controls. The primer sequences or IDs are shown in Supplemental Table 1. Gene expression in MCT and PBS-injected animals were compared using unpaired Student's t test.

Endogenous ELA Peptide Expression Localized by Immunostaining

Dual-labelling immunofluorescent staining was conducted as described[7] using ELA antiserum that cross reacted with ELA peptides, but not apelin (Supplemental Figure 1), the endothelial marker von-Willebrand factor (vWF) (1:50) and frozen sections of human histologically normal blood vessels (n=3-6), LV (n=8), lung (n=7), and primary PAECs (n=3). Peroxidase (DAB) stained, formaldehyde fixed human lung sections from idiopathic or familial PAH patients (n=10) and controls (n=10) were scored as positive or negative for ELA staining in

100 blood vessels with diameter ≤100μm. The average proportions of ELA-positive and negative vessels were compared between normal and PAH sections using Fisher's exact test.

Immunostaining was carried out as previously described[12]. ELA peptide expression was studied using rabbit polyclonal primary antiserum against human [pGlu^1]ELA-32 (Phoenix Pharmaceuticals, Belmont, CA, USA). An ELISA was performed to confirm cross-reactivity of the primary antiserum with ELA peptides but not apelin. Briefly, a 96-well plate with non-specific binding blocked by incubation with 3% bovine serum albumin was coated with 1μg/mL human ELA-32, ELA-21, ELA-11, or [Pyr^1]apelin-13 (or uncoated control) overnight at 4°C. Following washes with phosphate buffered saline 0.1% Tween-20 (PBS/T), polyclonal swine anti-rabbit IgG conjugated with horseradish peroxidase (Dako, Glostrup, Denmark) was applied for 2 hours at room temperature. After further washing, 3,3',5,5'-tetramethylbenzidine substrate was added for 5 minutes for generation of a colored product. The reaction was then quenched with 1mol/L sulfuric acid and the plate was read for absorbance at 450nm (ELx800 Absorbance Microplate Reader, BioTek, Winooski, VT, USA). Concentration response curves were fitted in GraphPad Prism 6.

For dual-label immunofluorescence with human tissues; 10μm fresh-frozen sections of human coronary, mammary, radial, and pulmonary artery (n=3 each) and 30μm fresh-frozen sections of human LV (n=8) and lung (n=7). Sections were fixed in acetone for 10 minutes. Non-specific binding was blocked by incubation with 5% goat serum in PBS for 2 hours at room temperature. Rabbit polyclonal primary antiserum against human [pGlu^1]ELA-32 (1:50 for vessels, 1:100 for LV, 1:500 for lung) was applied together with a monoclonal mouse anti-human von-Willebrand factor antibody (1:50) (Dako), used as a marker for endothelial cells, in PBS/T containing 3% goat serum on the sections for overnight incubation at 4°C. The primary antisera was omitted on adjacent negative control sections. Following 3 washes in cold PBS/T,

Alexa Fluor® 488 donkey anti-rabbit IgG (1:100) and Alexa Fluor® 568 donkey anti-mouse IgG (1:100) (both from Life Technologies) were applied in PBS/T containing 3% donkey serum and incubated for 2 hours at room temperature. This was followed by washing and mounting with ProLong Gold® antifade reagent (Life Technologies), and imaging using a Leica TCS SP8 confocal laser scanning microscope (Leica Microsystems, Milton Keynes, UK). To study the expression of ELA peptide in endothelial cells, PAECs (Lonza, Basel, Switzerland) were cultured in EGM™-2 medium with 2% serum, and plated on coverslips at passage 7 for immunostaining (n=3). The cells were fixed in methanol/acetone and the staining was conducted as above. All images were processed in the same method using Fiji[13, 14] for background subtraction using the rolling ball method, histogram stretching and merging of the channels.

ELA expression in blood vessels in normal and PAH human lung sections was investigated as previously described[15]. Peroxidase/DAB staining was carried out using human lung sections from idiopathic or familial PAH patients (n=10) and controls (n=10). Tissues were fixed with formaldehyde, cut into 10μm sections and processed for antigen retrieval using the Dako PT Link instrument according to manufacturer's instructions. Non-specific binding was blocked by incubation with 5% goat serum in PBS for 2 hours at room temperature. Rabbit polyclonal primary antiserum against human [pGlu^1]ELA-32 (1:500) in PBS/T containing 3% goat serum on the sections for overnight incubation at 4°C. Following 3 washes in cold PBS/T, sections were incubated for 1 hour with 1:100 goat anti-rabbit IgG antibody, washed again, and incubated for 1 hour with 1:200 rabbit PAP complex. After repeated washing in PBS/T, 3,3-diaminobenzidine in 0.1M Tris-HCl with 3% hydrogen peroxide was applied for 4 minutes. Then the tissues were dehydrated through an alcohol series and cleared in xylene, and mounted with DePeX medium (VWR International Ltd, Lutterworth, UK). Blood vessels with a diameter ≤100μm were scored blindedly as positive or negative for ELA staining at 375x

magnification on a Polyvar Met microscope (Reichert Technologies, Munich, Germany), with 100 vessels counted from each section. The average number of ELA-positive and negative vessels were compared between normal and PAH sections using Fisher's exact test in GraphPad Prism 6.

Enzyme Immunoassays

For detection of ELA peptide levels in plasma human blood was collected from healthy volunteers (n=25, 20 male and 5 female, age=30±2 years) into heparinised tubes and centrifuged at 2000xg for 5 minutes to extract plasma. Concentrations of apelin peptides were measured using a widely used enzyme immunoassay (EK-057-23, Phoenix Pharmaceuticals)[1, 16] and concentrations of ELA peptides were measured using a selective immunoassay from the same manufacturer (EK-007-19, Phoenix Pharmaceuticals), following manufacturer's instructions. Briefly, all samples were assayed in duplicates and diluted 1:2 in the assay buffer to minimise interference. Samples were added to wells of a secondary antibody-coated plate and incubated with primary antibody and competing biotinylated peptide for 2 hours at room temperature with orbital shaking. The wells were then washed and streptavidin-horseradish peroxidase added to incubate for an hour. Following another washing, the enzyme substrate solution was added and incubated for an hour. The reaction was quenched by adding hydrochloric acid and the plate was read on the plate reader for absorbance at 450nm. The absorbance was proportional to the amount of biotinylated peptide-peroxidase complex and therefore inversely proportional to the amount of peptied in the samples. The unknown concentration in samples were determined by extrapolation to a standard curve of known concentrations and multiplying by the dilution factor. The calculated concentrations of ELA and apelin were compared using Student's t test and tested for correlation using Pearson's test in GraphPad Prism 6.

For detection of changes in plasma cardiovascular peptides in response to ELA-32 administration, plasma from either ELA-32 or saline control treated rats was collected in EDTA tubes and centrifuged at 2000xg for 5 minutes to extract plasma. Angiotensin –II (Catalogue no. EKE-002-12) and BNP-32 (Catalogue no. EK-011-14) levels were measured using specific enzyme immunoassays according to the maufacturer's instructions (Phoenix Pharmaceuticals , Inc. CA, USA) as described above.

Effects of ELA and Apelin *in vivo*

The cardiac effects of ELA and apelin *in vivo* was first studied using magnetic resonance imaging (MRI), as previously described[17]. Male Sprague Dawley rats (264±2g) were anaesthetised with gaseous isoflurane both for induction (3% in 1.5 L/min oxygen) and maintenance (1.5-2.5% in 1.5 L/min oxygen). For compound administration, the right external jugular vein was cannulated with polyethylene tubing (Smiths Medical, Ashford, UK) filled with heparinised 0.9% saline solution. A pressure sensor for respiration rate was used to monitor depth of anaesthesia, with respiration rate was maintained at 45-55 breaths per minute. Body temperature was monitored using a rectal thermometer and maintained at 37°C using a flowing-water heating blanket. Prospective gating of the MRI sequences was achieved with electrocardiography monitoring using paediatric ECG electrodes (3M Europe, Diegem, Belgium) on left and right forepaws. MRI was performed at 4.7 T with a Bruker BioSpec 47/40 system (Bruker Inc., Ettlingen, Germany). A birdcage coil of 12cm was used for signal excitation and animals were positioned prone over a 2cm surface coil for signal reception. After initial localisation images, 4-chamber and 2-chamber views were obtained. Using these scans as a reference, short axis slices were acquired (FISP, TR/TE 6 ms/2.1 ms, 20-30 frames, 5 cm FOV, 256x256 matrix, 2 mm slice thickness, bandwidth 78 kHz, flip angle 20°, NEX 2), perpendicularly to both the long-axis views. Full LV and RV coverage in the short axis was achieved with no slice gap with 9-10 slices. After acquiring this reference baseline scan, ELA-

32 (20 and 150nmol, n=8), [Pyr[1]]apelin-13 (50 and 650nmol, n=5) or the same volume (500µl) of saline (n=6) was administered as two cumulative bolus intravenous injections through the implanted cannula and flushed with 100µL saline. Following the injections, three mid-ventricular slices were planned in the short axis of the heart in order to capture changes in function with a 1.5 min resolution. (FISP, TR/TE 6 ms/2.1 ms, 20-30 frames, 5 cm FOV, 128x128 matrix, 2 mm slice thickness, bandwidth 78 kHz, flip angle 20°, NEX 1). The effects of the compounds were monitored for no less than 10 minutes. Delineation of the LV and RV was as described using Segment v1.9[17, 18]. Measurements made from the three slices were normalised to the baseline scan to estimate the volumes at end-systolic and end-diastolic points. The LV and RV ejection fraction was calculated and expressed as change from baseline. The peak effects induced by ELA, apelin and saline were tested using one-way ANOVA with Dunnett's post-test comparing ELA and apelin groups to the saline group using GraphPad Prism 6.

The MRI study of the *in vivo* cardiac effects of ELA and apelin was complemented by catheterization experiments. The surgical part of the experiment was performed as previously described[19]. Male Sprague Dawley rats (257±7g) anaesthetised with gaseous isoflurane (3% for induction and 1.5-2% for maintenance, 1.5L/min oxygen). Body temperature was monitored using a rectal thermometer and maintained at 37°C. For compound administration, the right external jugular vein was cannulated with polyethylene tubing (Smiths Medical) filled with heparinised 0.9% saline solution. A pressure volume catheter (SPR-869, Millar, ADIstruments, Oxford, UK) was connected to the data PowerLab 16/35 system with LabChart 8 (ADIstruments, Oxford, UK) and calibrated using the MPVS Ultra PV Unit (ADIstruments, Oxford, UK). Then the catheter was inserted into the LV via the right carotid artery. The position of the catheter was determined by the blood pressure and shape of the pressure volume loops. The animal was allowed to stabilise for recording of baseline hemodynamic parameters.

ELA-32 (20 and 150nmol, n=10), [Pyr[1]]apelin-13 (50, 400 and 1300nmol, n=8) or the equal volume (500µl) of saline (n=6) were given as cumulative bolus intravenous injections via the cannula, which was flushed with 100µL saline. The effects of the compounds were monitored for no less than 10 minutes. Data analysis was performed using LabChart 8 as previously described[20]. The peak effects of ELA, apelin and saline on LV systolic pressure, cardiac output, contractility (dP/dt_{MAX}) were expressed as change from baseline and tested using one-way ANOVA with Dunnett's post-test comparing ELA and apelin groups to the saline group using GraphPad Prism 6.

Monocrotaline-Induced Rat Model of PAH

Methods were as previously described[21]. Male Sprague-Dawley rats (205±2g) were randomly allocated to receive MCT (n=18; 60mg/kg body weight) or an equal volume of vehicle (n=17; 0.9% saline) by subcutaneous injection on day 0. From day 1 to day 21, randomly selected MCT (n=9) and vehicle control (n=9) exposed animals received daily intraperitoneal injections of ELA-32 (450µg/kg body weight) with the remainder (MCT, n=9; saline, n=8) receiving intrperitoneal injections of saline for 21 days. On day 21, the rats were weighed and catheterized for right ventricular hemodynamic measurements as previously described[21, 22]. Briefly, rats were anaesthetized with gaseous isoflurane (3% for induction, 1.5-2.5% for maintenance, 1.5 L/min oxygen). A pressure volume catheter (SPR-869, Millar) connected to the data PowerLab 16/35 system with LabChart 5 and calibrated using the MPVS Ultra PV Unit was inserted into the right ventricle via the right external jugular vein to measure right ventricular systolic pressure (RVSP). To investigate any effect of chronic ELA administration on systemic blood pressure, carotid artery and LV catheterization was performed in ELA control and saline control animals (n=5 each group) as described above. The position of the catheter was determined by the blood pressure and shape of the pressure volume loops. Then the rats were euthanized by exsanguination, terminal blood samples were collected into EDTA

tubes for determination of plasma levels of angiotensin-II and BNP-32. The left lung infused with 0.8% agarose to inflate, removed, fixed in 10% formalin (CellPath, Powys, UK), paraffin embedded and stained for smooth muscle α-actin and elastic van Giesen as described[21, 22]. Other tissues collected included the heart, the RV was dissected from the LV+septum and the weight ratio of these (RV compared to LV+septum), also known as the Fulton index, was determined as a measure of right ventricular hypertrophy. Paraffin embedded sections of RV were compared for evidence of cardiomyocyte hypertrophy or proliferation by measuring cardiomyocyte cross-sectional area of at least 100 cardiomyocytes in at least six myocardial zones per section stained with FITC conjugated WGA (wheat germ agglutinin) using 'threshhold and analyze particle' tool in ImageJ. Additionally, the average number of cardiomyocyte nuclei/area and number of GATA4 positive nuclei/area[23] were counted in five different myocardial zones. In order to compare size and numbers of cells accurately between zones and between tissue sections, only cells in one particular orientation were included. In the left lung small (diameter 25-75μm) pulmonary blood vessels associated with alveolar ducts were scored as completely muscular, partially muscular, or non-muscular with 100 vessels scored from each section. Statistical significance was assessed by comparing the percentage of fully muscularized vessels between groups. The wall thickness of pulmonary arteries (diameter >75μm) close to terminal bronchioles was determined by measuring the average wall thickness as a percentage of average lumen diameter of the vessel using ImageJ as previously described[21]. Group data were compared using one-way ANOVA with Tukey's post-test.

Materials

Human [Pyr[1]]apelin-13 were synthesized by Severn Biotech (Kidderminster, UK). Human ELA-32 (the [pGlu[1]]ELA-32 form) was synthesized by Severn Biotech or purchased from Phoenix Pharmaceuticals. Human ELA-21 and ELA-11 were from Phoenix Pharmaceuticals (Belmont, CA, USA). ELA-14 and cyclo[1-6]ELA-11 were synthesized by Department of

Chemistry, University of Cambridge. All other reagents were from Sigma-Aldrich Ltd (Poole, UK), unless otherwise stated.

Supplemental Table

Supplemental Table 1: Primer sequences or IDs for RT-qPCR.

Target	Primer Sequences	
Human *APELA*	Sense	GAAGAAGAAGAGGAGTGAAGGA
	Antisense	CCATTCCAGGTGCTTTCAAAT
Rat *Apela*	Sense	AGTCACTGATCTCCTTGGTTACC
	Antisense	CTGCCGCACTGTTGCCA
Primers from Thermofisher		
Target	Primer Assay ID	
Human 18S rRNA	Hs99999901_s1	
Rat 18S rRNA	Rn03928990_g1	
Rat *Aplnr*	Rn00580252_s1	
Rat *Apln*	Rn00581093_m1	

Supplemental Results and Figures

Cross-Reactivity Testing of the Human ELA Antiserum

As expected, the antiserum showed similar cross-reactivity to human ELA-32, ELA-21 and ELA-11 peptides but did not cross-react with human [Pyr^1]apelin-13 peptide (Supplemental Figure 1). Therefore it can be used as an ELA-specific antiserum.

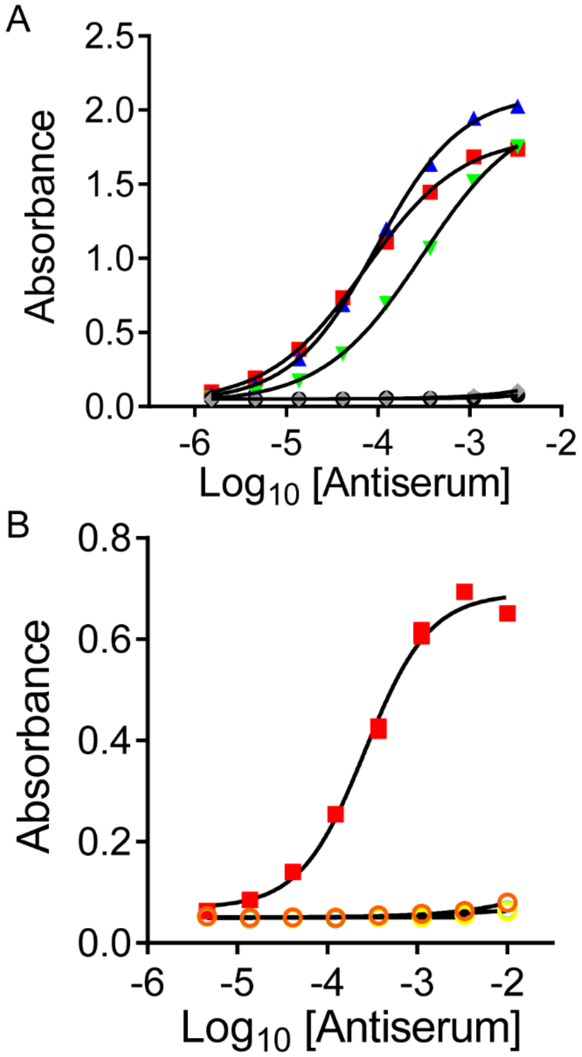

Supplemental Figure 1. Custom ELISA confirms cross-reactivty and specificity of the ELA antibody. Cross reactivity to (A) ELA-32 (■), ELA-21 (▲), ELA-11 (▼), but not to [Pyr^1]apelin-13 (●) or uncoated controls (◆). (B) Compared to ELA (■), no cross reactivity was obtained with other cardiovascular peptides; angiotensin II (○), bradykinin (○), endothelin-1 (○).

Effect of [Pyr[1]]Apelin-13 and ELA-32 on Levels of Protein Phosphorylation in PAEC and PASMCs *in vitro*

There were significant increases in phosphorylation levels of ERK1/2 and eNOS in response to [Pyr[1]]apelin-13 (100nmol/L) or ELA-32 (100nmol/L) in cultured PAECs.

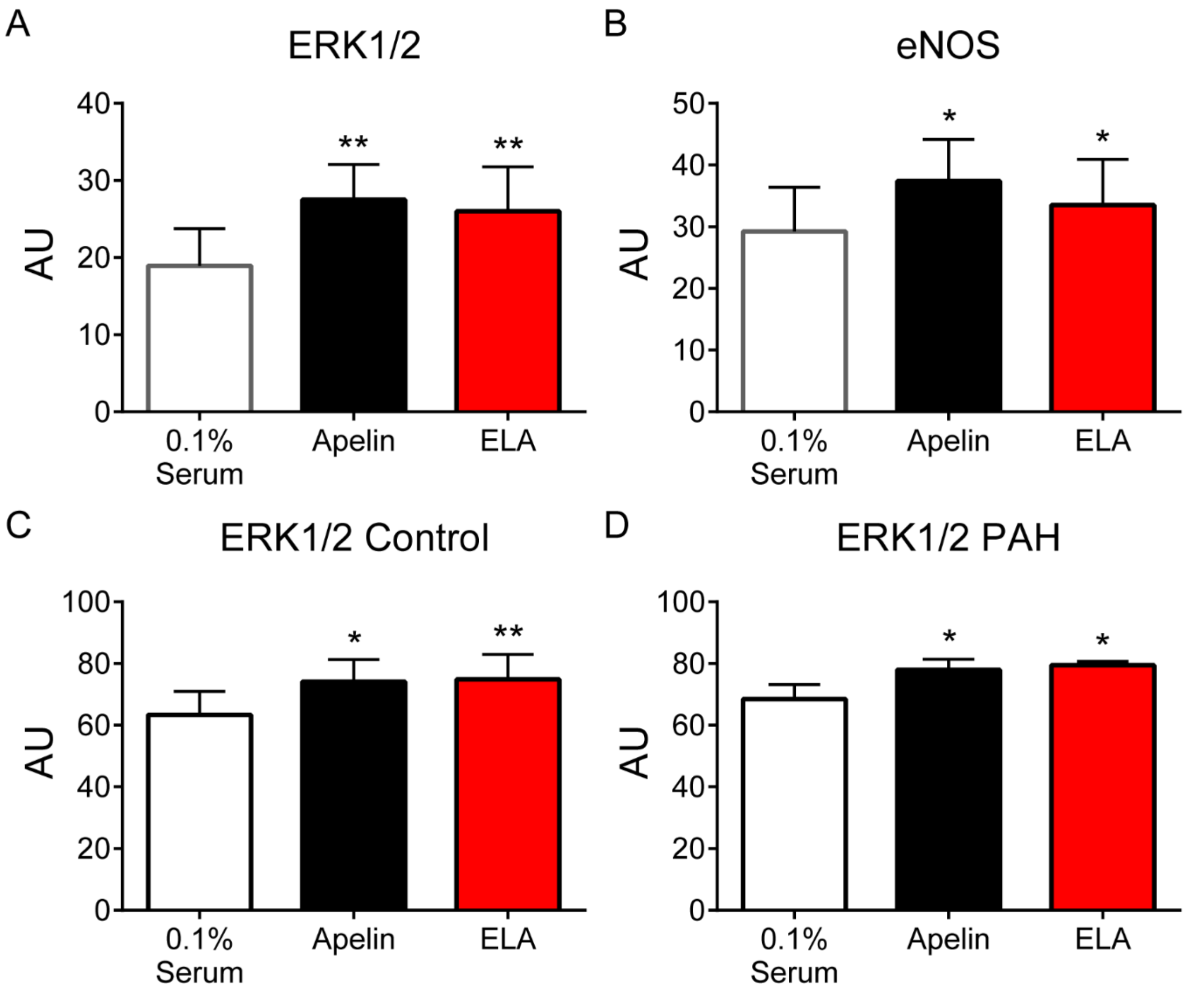

Supplemental Figure 2. Effect of [Pyr[1]]Apelin-13 and ELA-32 on levels of protein phosphorylation in PAEC and PASMCs *in vitro*. Increased phosphorylation levels of (A) ERK1/2 and (B) eNOS in PAECs and ERK1/2 in (C) control and (D) PAH PASMcs following treatment with 0.1% serum (control), [Pyr[1]]apelin-13 or ELA-32 (both 100nmol/L). Significantly different from control * P≤0.05, **P≤0.01, one-way ANOVA for repeated measures with Tukey's post-test for multiple comparisons.

There was no significant effect on level of phosphorylation of either peptide on an additional 41 kinases and 2 total proteins (Supplemental Figure 3).

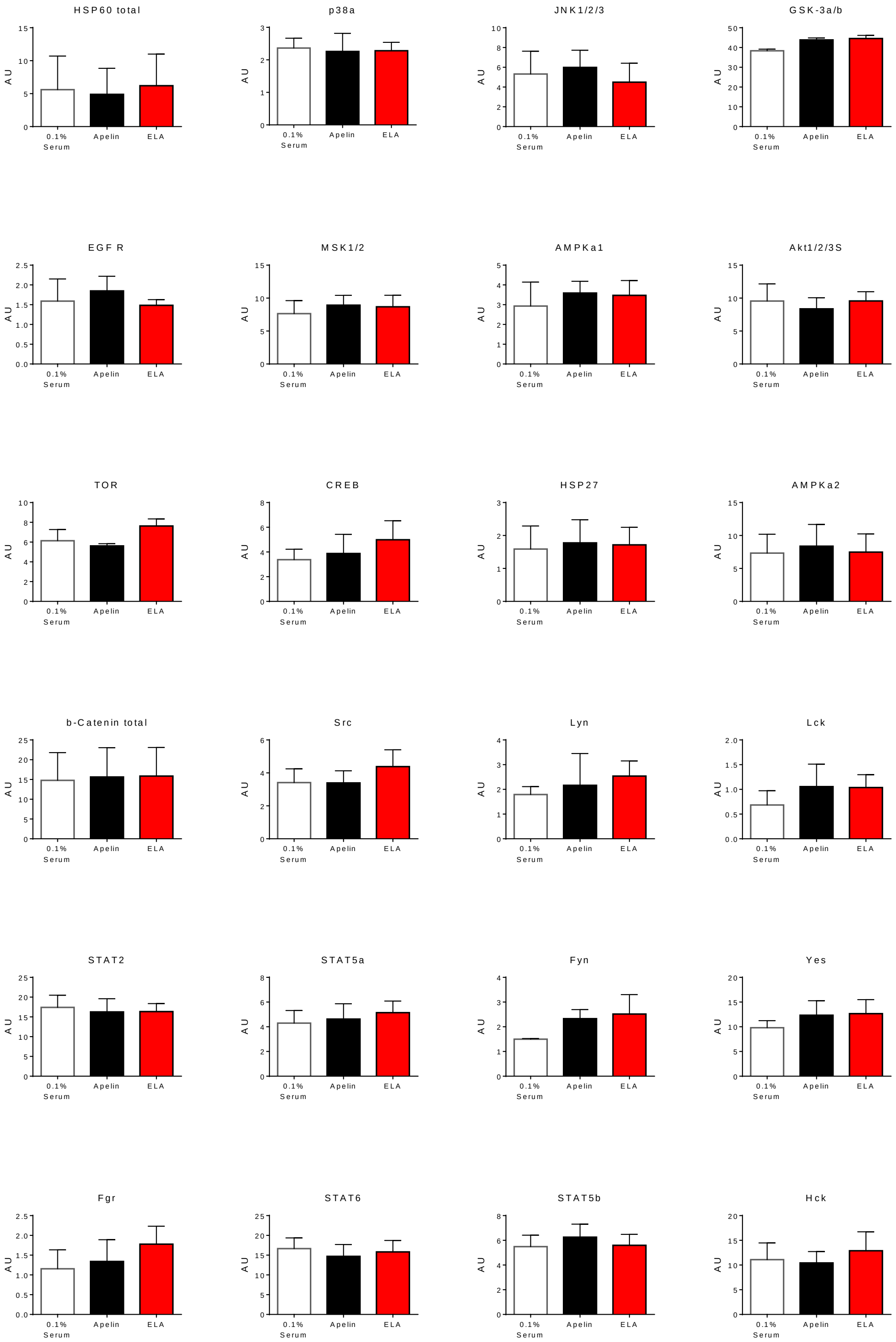

Supplemental Figure 3. Lack of effect of [Pyr^1]apelin-13 and ELA-32 on protein phosphorylation levels.

Supplemental Figure 3 continued.

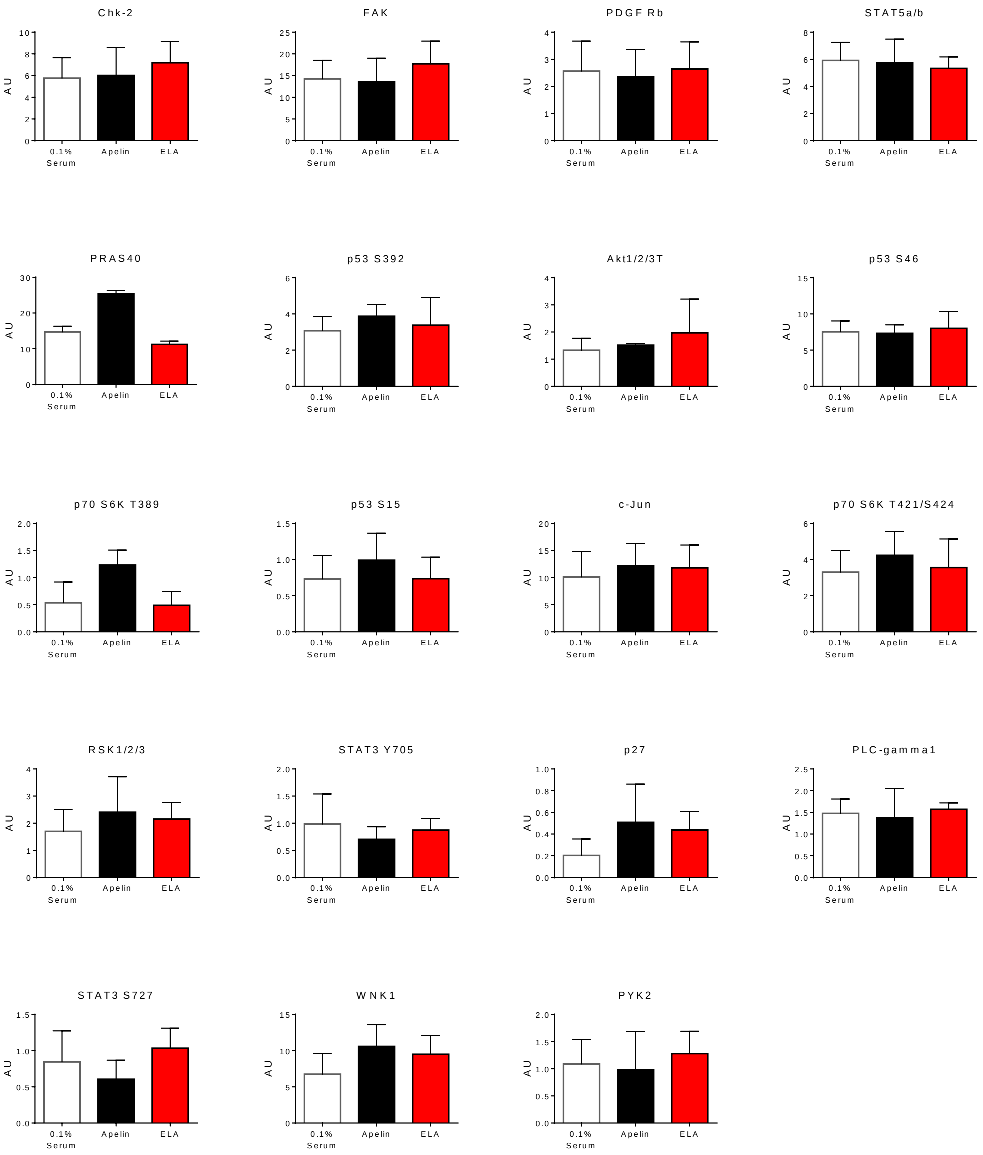

There were no significant changes in levels of secreted angiogenesis factors in response to [Pyr^1]apelin (100nmol/L) or ELA-32 (100nmol/L) treatment in either PAECs (Supplemental Figure 4) or control/PAH PASMCs (Supplemental Figure 5).

Supplemental Figure 4. Lack of effect of [Pyr[1]]Apelin-13 and ELA-32 on levels of secreted angiogenesis factors in cultured PAECs.

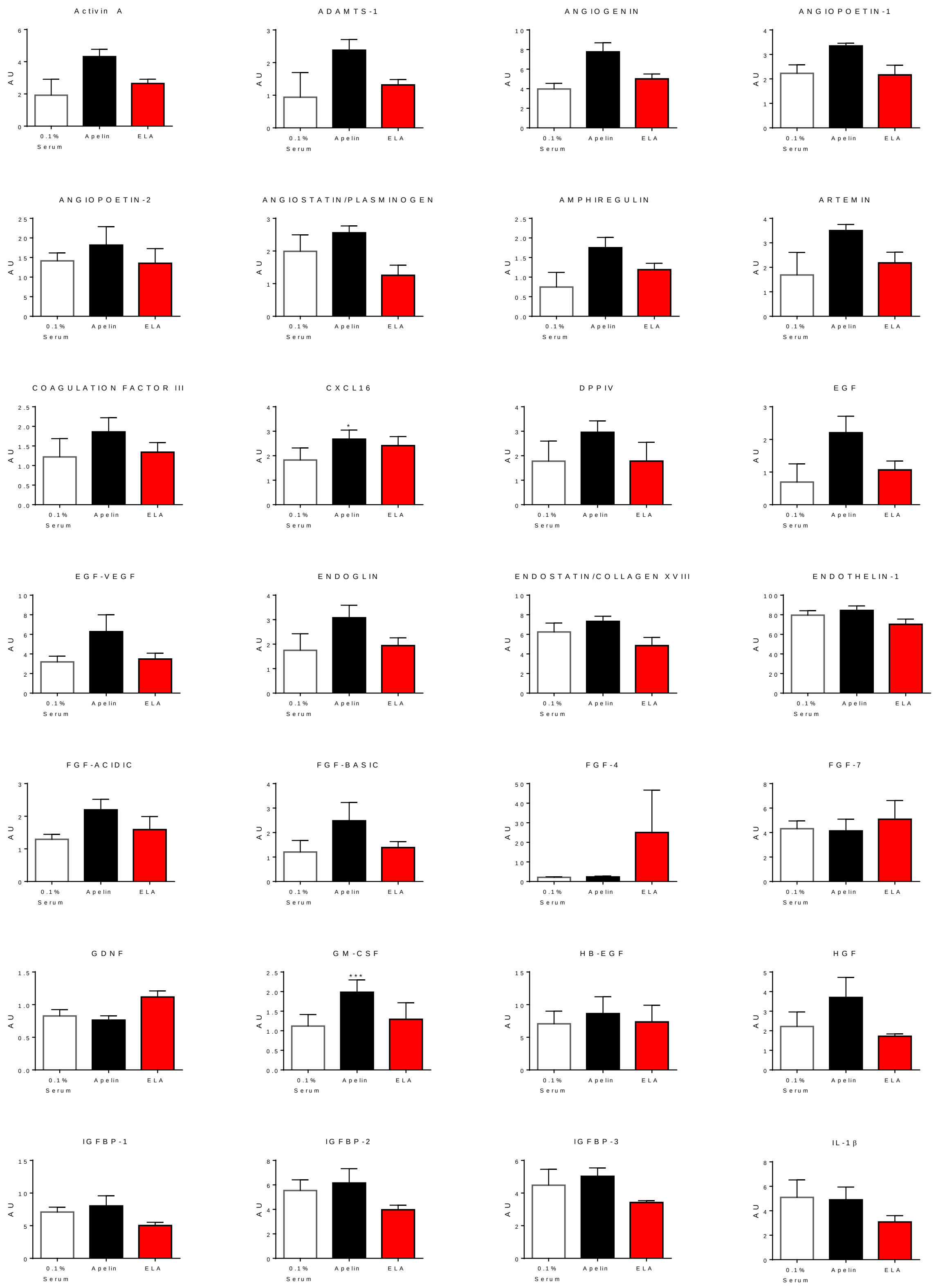

Supplemental Figure 4 continued

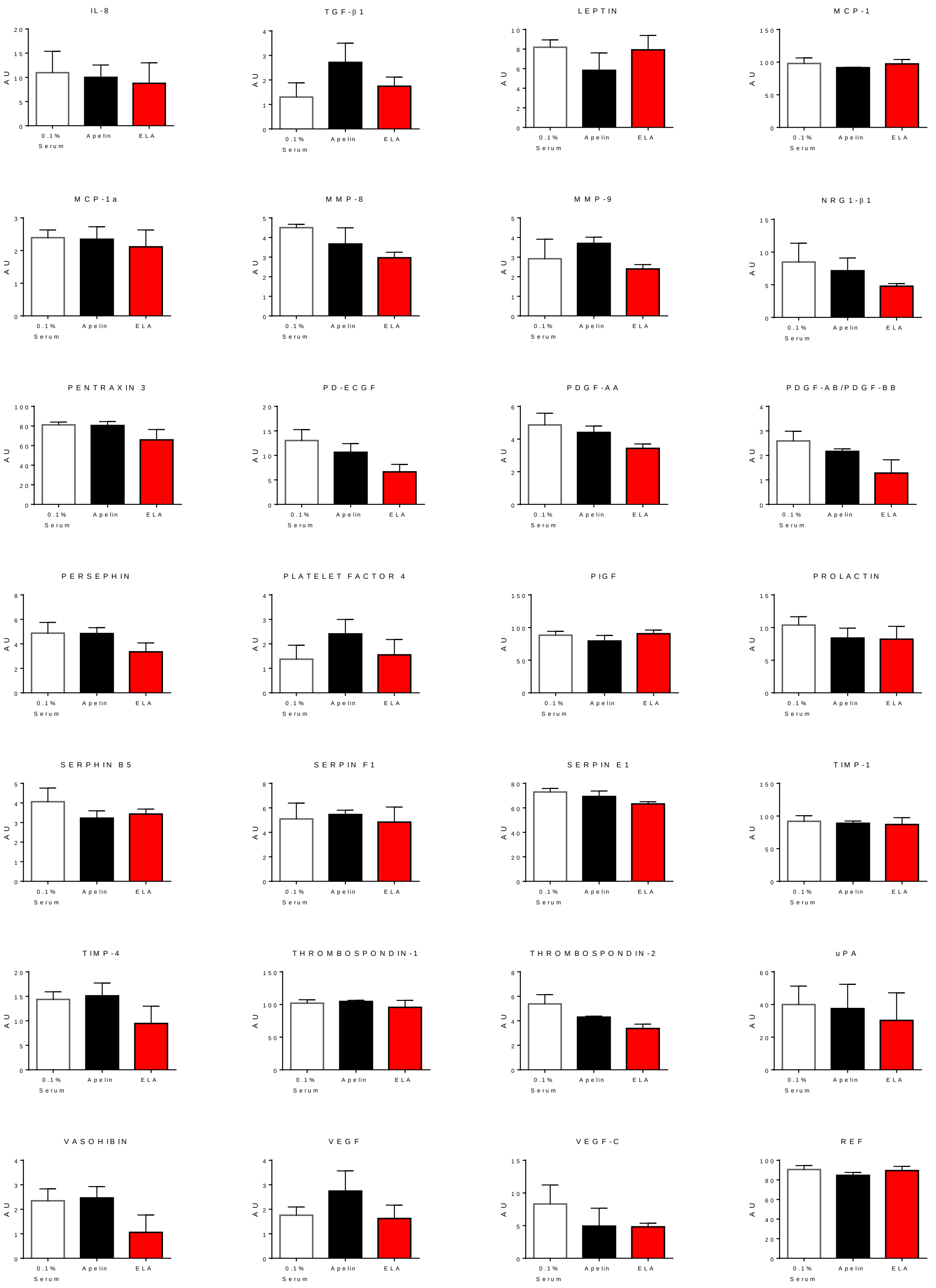

Supplemental Figure 5. Lack of effect of [Pyr[1]]Apelin-13 and ELA-32 on levels of secreted angiogenesis factors in cultured control and PAH PASMCs.

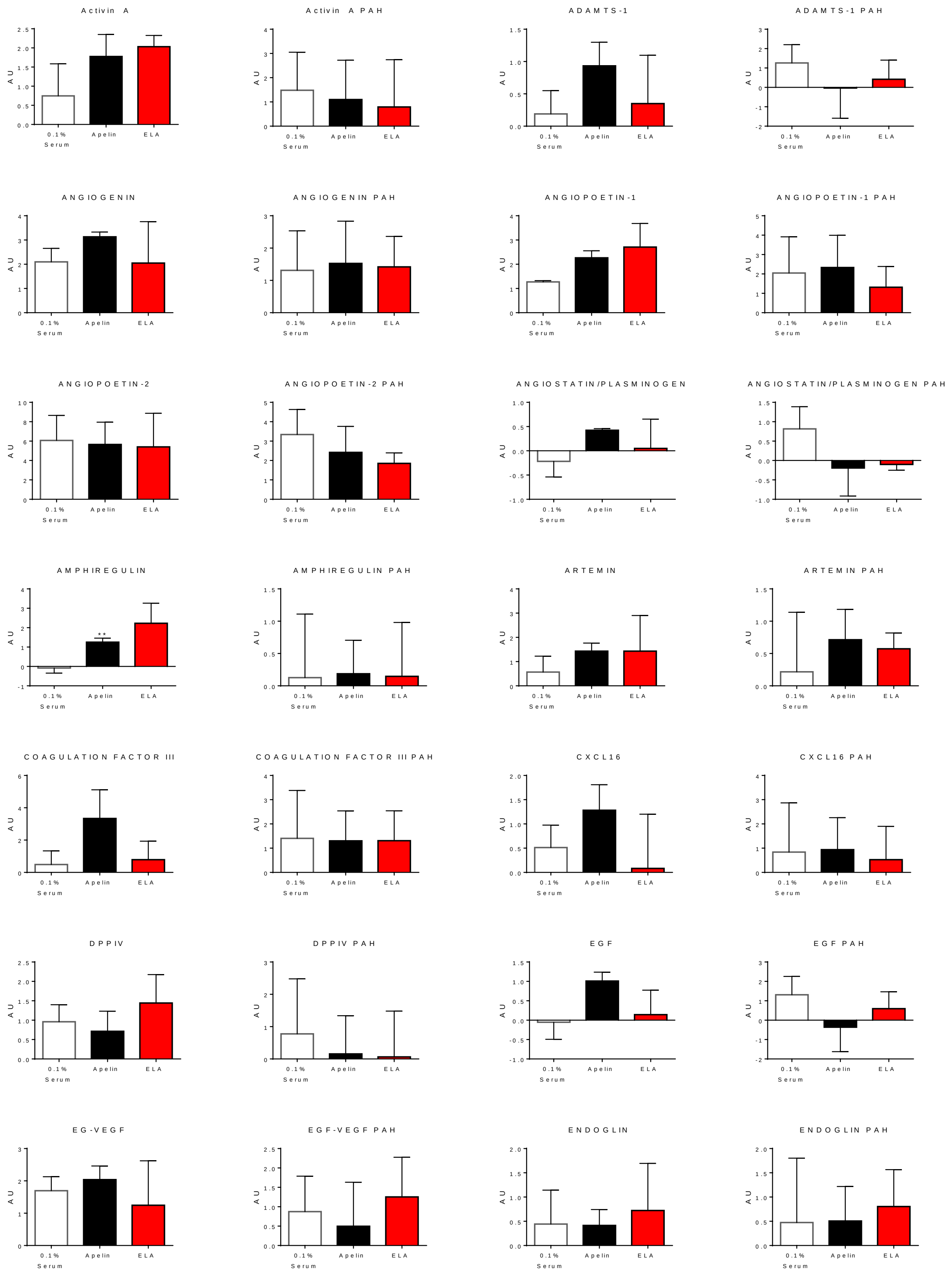

Supplemental Figure 5 continued.

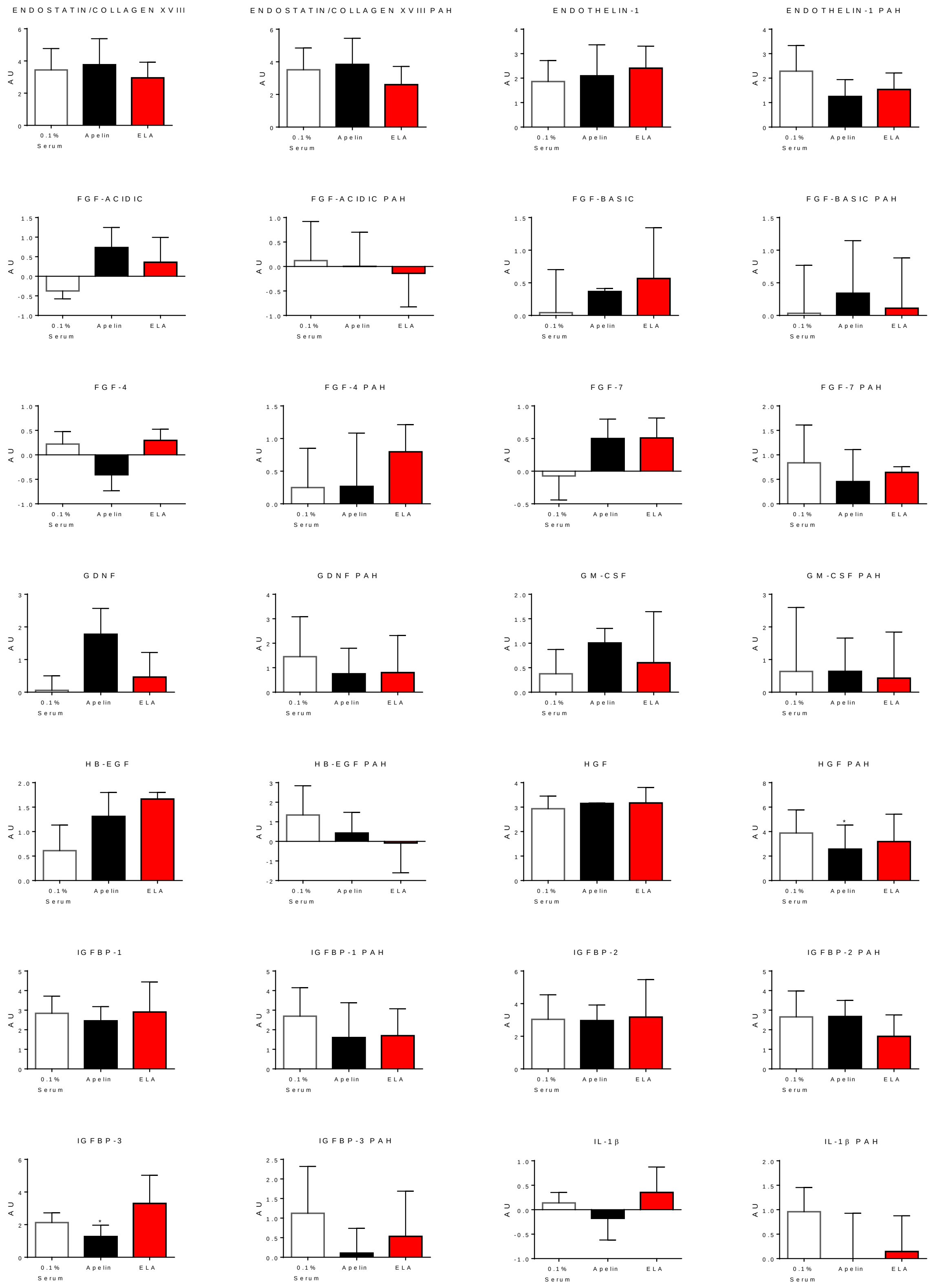

Supplemental Figure 5 continued.

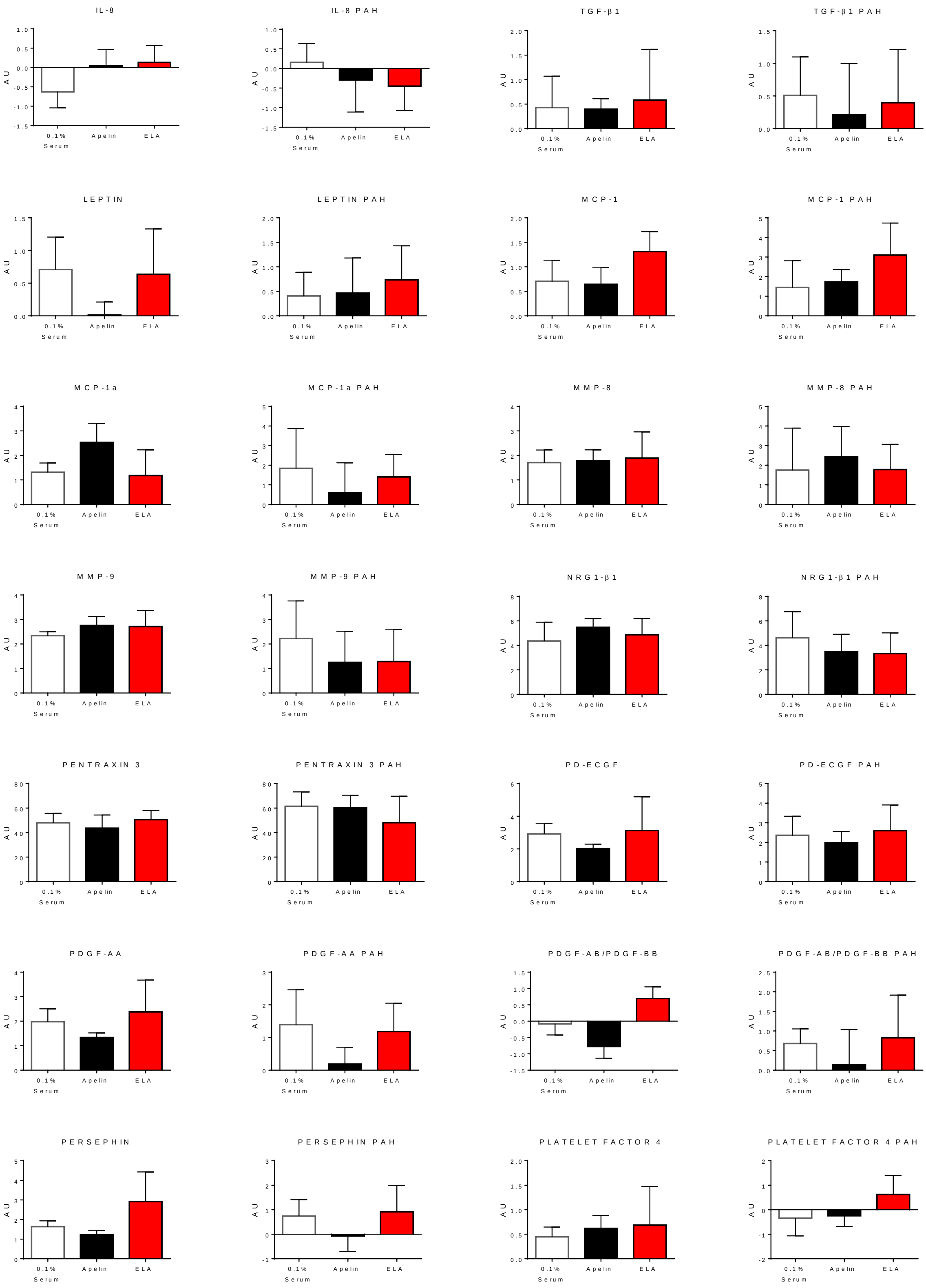

Supplemental Figure 5 continued.

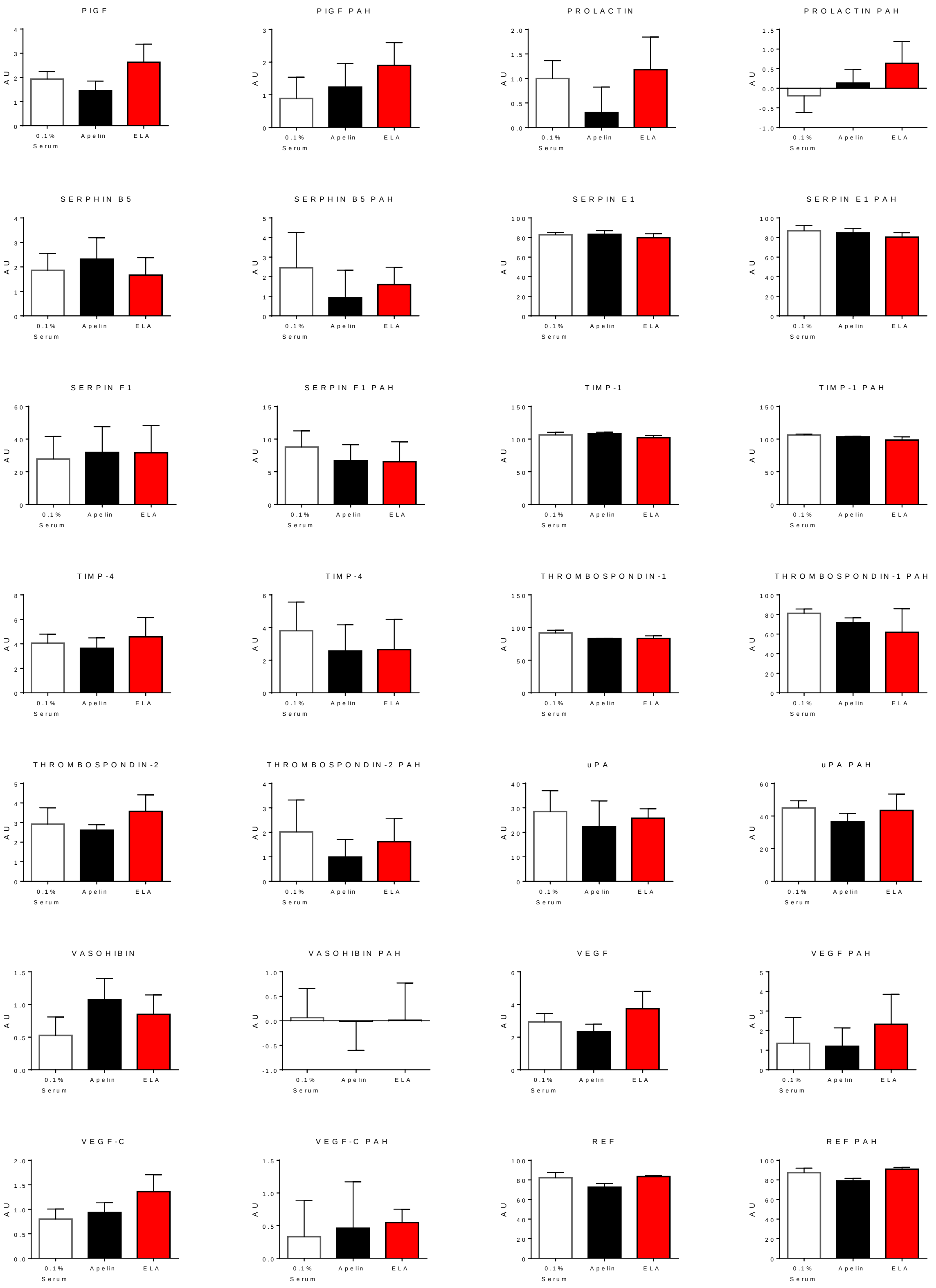

Expression of ELA in Human PAECs and Endothelium of Human Cardiovascular Tissues

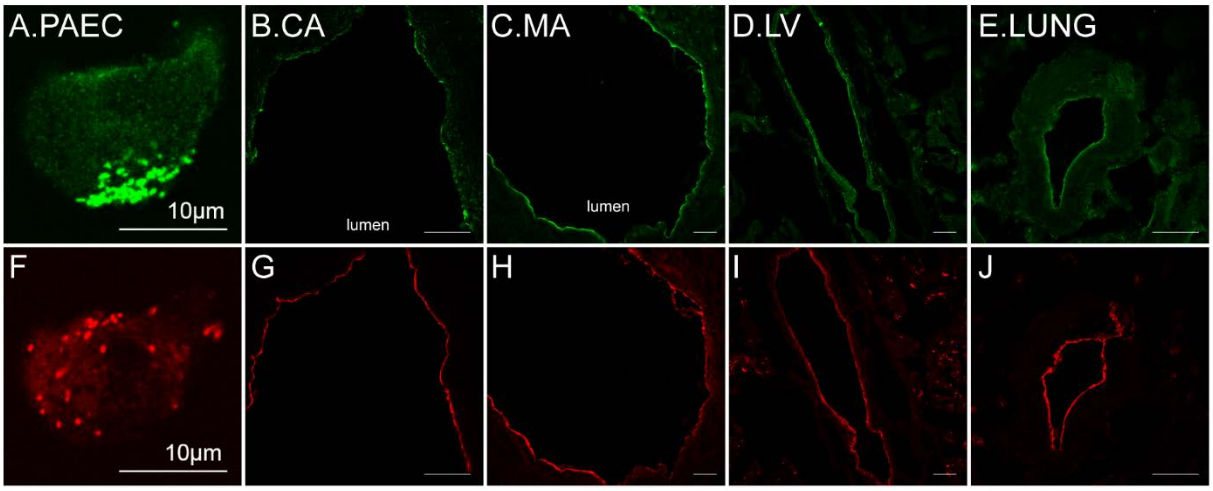

Supplemental Figure 6. Expression of ELA in human PAECs and endothelium of human cardiovascular tissues. Immunocytochemical localization of ELA-like immunoreactivity (green fluorescence) and vWF-like immunoreactivity in human (A, F) PAECs and endothelium of human (B, G), coronary artery (CA), (C, H) mammary artery (MA), (D, I) left ventricle (LV), (F, J) lung sections. Scale bars=75µm unless indicated.

Effects of ELA *in vivo*

There was no significant effect of ELA-32 and [Pyr^1]apelin-13 on heart rate in rat *in vivo*

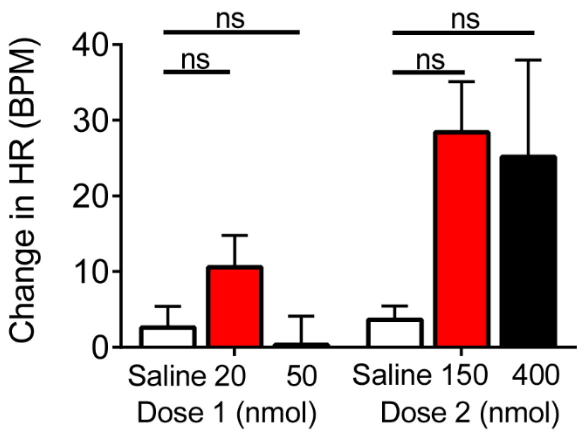

Supplemental Figure 7 The *in vivo* effects of increasing doses of ELA-32 (red bars) and [Pyr1]apelin-13 (black bars) on heart rate compared to saline controls (open bars). BPM, beats per minute.

Reduced Endothelial Staining of ELA in PAH Human Lung

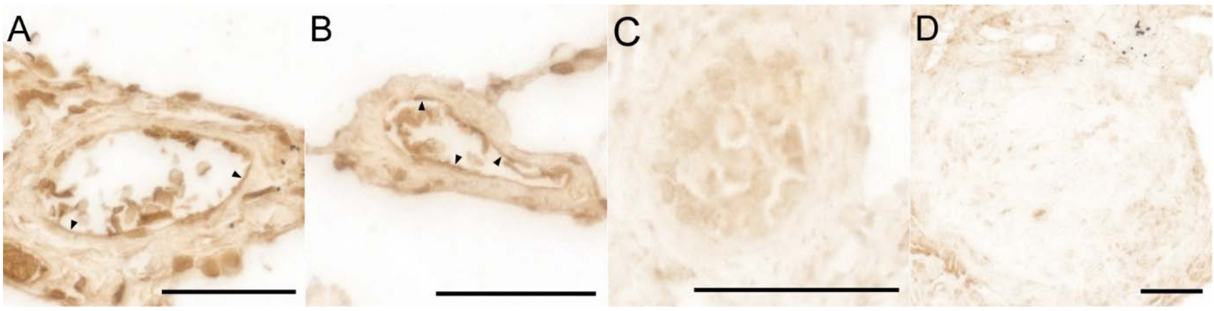

Supplemental Figure 8. Reduced endothelial ELA expression in human PAH lung. (A and B) Representative microphotographs of ELA-positive blood vessels (staining indicated by arrow heads) in a normal lung section. (C) Representative microphotograph showing ELA-negative blood vessels in a PAH lung section. (D) Representative microphotograph showing the absence of ELA-like immunoreacitivity in a pathological vascular lesion in a PAH lung section. Some non-specific staining due to red blood cell peroxidase are present as a useful marker of blood vessels. Scale bar=50µm.

Attenuation of Right Ventricular Hypertrophy by ELA in MCT Exposed Rat Heart

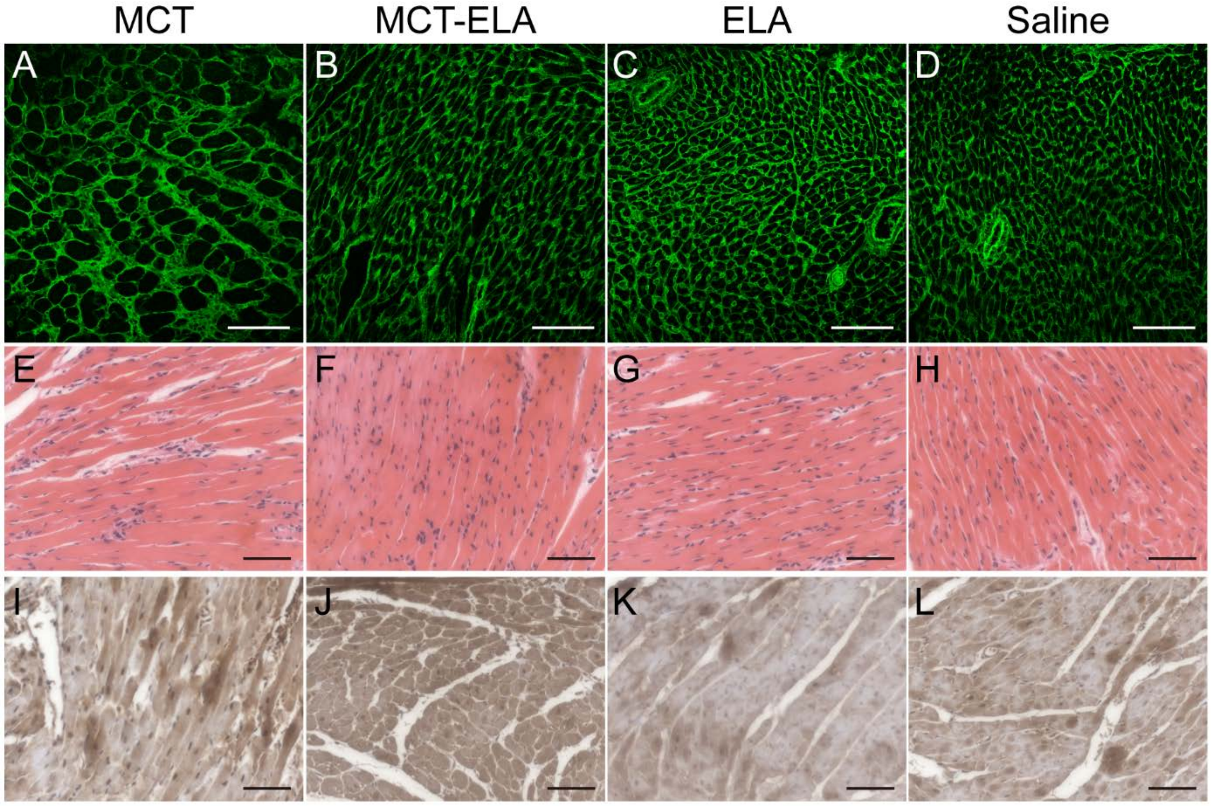

Supplemental Figure 9. Attenuation of right ventricular hypertrophy by ELA in MCT exposed rat heart. MCT exposure also resulted in significant RV hypertrophy indicated by (A) an increase in cardiomyocyte are (WGA staining), (E) a reduction in cardiomyocyte number/area (hematoxylin and eosin staining) and (I) an increase in the number of GATA4 positive nuclei/area compared to saline controls (D, H. L). There was a significant improvement in these following ELA-32 treatment of MCT exposed rats (B, F, J). ELA alone (C, G, K) had no significant effect compared to saline control. Scale bar = 75μm.

Lack of Significant Effect of Chronic Administration of ELA on Systemic Blood Pressure

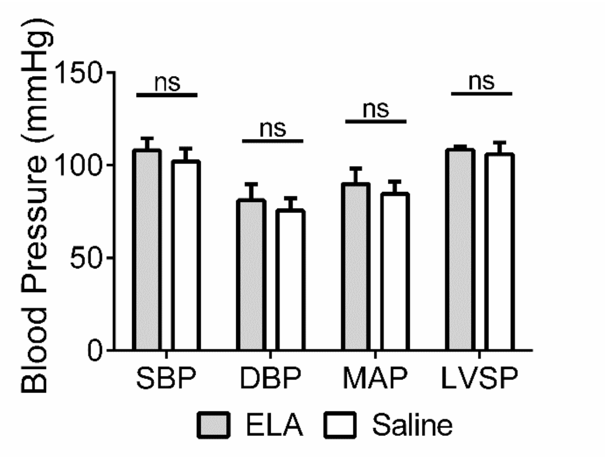

Supplemental Figure 10. Lack of significant effect of chronic administration of ELA on systemic blood pressure. 21 Days of ELA administration (n=5) did not affect systolic blood pressure (SBP), diastolica blood pressure (DBP), mean arterial pressure (MAP) or left ventricular systolic pressure (LVSP) compared to saline control (n=5).

No Significant Effect of ELA-32 Treatment on Plasma Levels of Angiotensin-II or BNP-32 Levels in Saline Control and MCT Exposed Rats.

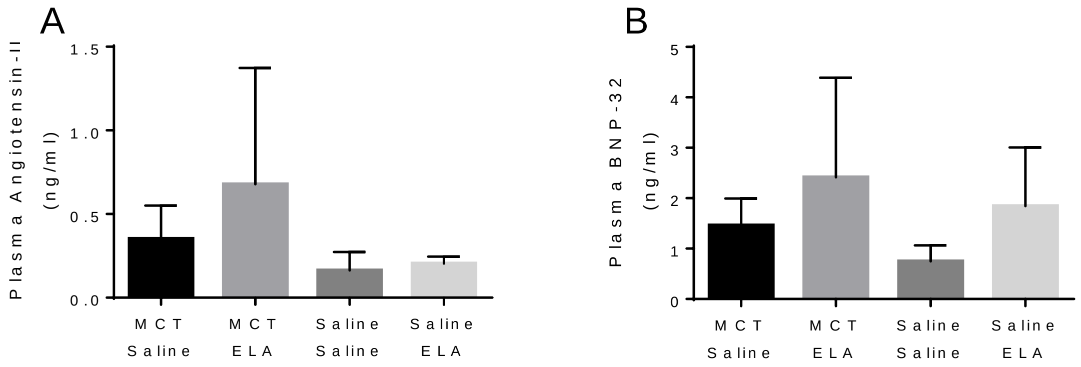

Supplemental Figure 11. Plasma levels of (A) angiotensin-II and (B) BNP-32 were not significantly affected by treatment with ELA-32 for 21 days in saline control or MCT exposed rats.

Supplemental References

1. Brame AL, Maguire JJ, Yang P, Dyson A, Torella R, Cheriyan J, Singer M, Glen RC, Wilkinson IB, Davenport AP. Design, characterization, and first-in-human study of the vascular actions of a novel biased apelin receptor agonist. *Hypertension*. 2015;65:834-840.
2. Wu B, Chien EY, Mol CD, Fenalti G, Liu W, Katritch V, Abagyan R, Brooun A, Wells P, Bi FC, Hamel DJ, Kuhn P, Handel TM, Cherezov V, Stevens RC. Structures of the CXCR4 chemokine GPCR with small-molecule and cyclic peptide antagonists. *Science.* 2010;330:1066-1071.
3. Sali A, Blundell TL. Comparative protein modelling by satisfaction of spatial restraints. *J Mol Biol.* 1993;234:779-815.
4. Sastry GM, Adzhigirey M, Day T, Annabhimoju R, Sherman W. Protein and ligand preparation: Parameters, protocols, and influence on virtual screening enrichments. *J Comput Aided Mol Des.* 2013;27:221-234.
5. Jones G, Willett P, Glen RC. Molecular recognition of receptor sites using a genetic algorithm with a description of desolvation. *J Mol Biol.* 1995;245:43-53.
6. Jones G, Willett P, Glen RC, Leach AR, Taylor R. Development and validation of a genetic algorithm for flexible docking. *J Mol Biol.* 1997;267:727-748.
7. Iturrioz X, Gerbier R, Leroux V, Alvear-Perez R, Maigret B, Llorens-Cortes C. By interacting with the C-terminal Phe of apelin, Phe255 and Trp259 in helix VI of the apelin receptor are critical for internalization. *J Biol Chem.* 2010;285:32627-32637.
8. Murza A, Sainsily X, Coquerel D, Côté J, Marx P, Besserer-Offroy É, Longpré JM, Lainé J, Reversade B, Salvail D, Leduc R, Dumaine R, Lesur O, Auger-Messier M, Sarret P, Marsault É. Discovery and structure-activity relationship of a bioactive fragment of elabela that modulates vascular and cardiac functions. *J Med Chem.* 2016;59:2962-2972.

9. Clark AM, Labute P, Santavy M. 2D structure depiction. *J Chem Inf Model.* 2006;46:1107-1123

10. Maloney PR, Khan P, Hedrick M, Gosalia P, Milewski M, Li L, Roth GP, Sergienko E, Suyama E, Sugarman E, Nguyen K, Mehta A, Vasile S, Su Y, Stonich D, Nguyen H, Zeng FY, Novo AM, Vicchiarelli M, Diwan J, Chung TD, Smith LH, Pinkerton AB. Discovery of 4-oxo-6-((pyrimidin-2-ylthio)methyl)-4h-pyran-3-yl 4-nitrobenzoate (ML221) as a functional antagonist of the apelin (APJ) receptor. *Bioorg Med Chem Lett.* 2012;22:6656-6660.

11. Schmittgen TD, Livak KJ. Analyzing real-time PCR data by the comparative C(T) method. *Nat Protoc.* 2008;3:1101-1108.

12. Kleinz MJ, Davenport AP. Immunocytochemical localization of the endogenous vasoactive peptide apelin to human vascular and endocardial endothelial cells. *Regul Pept.* 2004;118:119-125.

13. Schneider CA, Rasband WS, Eliceiri KW. NIH image to ImageJ: 25 years of image analysis. *Nat Methods.* 2012;9:671-675.

14. Schindelin J, Arganda-Carreras I, Frise E, Kaynig V, Longair M, Pietzsch T, Preibisch S, Rueden C, Saalfeld S, Schmid B, Tinevez JY, White DJ, Hartenstein V, Eliceiri K, Tomancak P, Cardona A. Fiji: An open-source platform for biological-image analysis. *Nat Methods.* 2012;9:676-682.

15. Moseley EL, Atkinson C, Sharples LD, Wallwork J, Goddard MJ. Deposition of C4d and C3d in cardiac transplants: A factor in the development of coronary artery vasculopathy. *J Heart Lung Transplant.* 2010;29:417-423.

16. Chandra SM, Razavi H, Kim J, Agrawal R, Kundu RK, de Jesus Perez V, Zamanian RT, Quertermous T, Chun HJ. Disruption of the apelin-APJ system worsens hypoxia-induced pulmonary hypertension. *Arterioscler Thromb Vasc Biol.* 2011;31:814-820.

17. Buonincontri G, Methner C, Carpenter TA, Hawkes RC, Sawiak SJ, Krieg T. MRI and PET in mouse models of myocardial infarction. *J Vis Exp.* 2013;e50806:1-9.

18. Heiberg E, Sjögren J, Ugander M, Carlsson M, Engblom H, Arheden H. Design and validation of Segment--freely available software for cardiovascular image analysis. *BMC Med Imaging.* 2010;10:1-13.

19. Pacher P, Nagayama T, Mukhopadhyay P, Bátkai S, Kass DA. Measurement of cardiac function using pressure-volume conductance catheter technique in mice and rats. *Nat Protoc.* 2008;3:1422-1434.

20. LaCroix C, Freeling J, Giles A, Wess J, Li YF. Deficiency of M2 muscarinic acetylcholine receptors increases susceptibility of ventricular function to chronic adrenergic stress. *Am J Physiol Heart Circ Physiol.* 2008;294:H810-820.

21. Long L, Ormiston ML, Yang X, Southwood M, Gräf S, Machado RD, Mueller M, Kinzel B, Yung LM, Wilkinson JM, Moore SD, Drake KM, Aldred MA, Yu PB, Upton PD, Morrell NW. Selective enhancement of endothelial BMPR-II with BMP9 reverses pulmonary arterial hypertension. *Nat Med.* 2015;21:777-785.

22. Crosby A, Soon E, Jones FM, Southwood MR, Haghighat L, Toshner MR, Raine T, Horan I, Yang P, Moore S, Ferrer E, Wright P, Ormiston ML, White RJ, Haight DA, Dunne DW, Morrell NW. Hepatic Shunting of Eggs and Pulmonary Vascular Remodeling in Bmpr2(+/-) Mice with Schistosomiasis. *Am J Respir Crit Care Med.* 2015;192:1355-1365.

23. Oka T, Maillet M, Watt AJ, Schwartz RJ, Aronow BJ, Duncan SA, Molkentin JD. Cardiac-specific deletion of Gata4 reveals its requirement for hypertrophy, compensation, and myocyte viability. *Circ Res.* 2006;98:837-845.

Supplemental Video 1: The effect of ELA-32 on the rat heart *in vivo*. Mid-ventricular transverse view acquired using MRI with first video was taken at baseline and six subsequent time points within 10 minutes following the intravenous injection of ELA-32.

Supplemental Video 2: The effect of [Pyr^1]apelin-13 on the rat heart *in vivo*. Mid-ventricular transverse view acquired using MRI with first video was taken at baseline and six subsequent time points within 10 minutes following the intravenous injection of [Pyr^1]apelin-13.
